# Photocurable injectable Janus hydrogel with minimally invasive delivery for all-in-one treatment of gastric perforations and postoperative adhesions

**DOI:** 10.7150/thno.87639

**Published:** 2023-09-25

**Authors:** Xiaoqi Wu, Zihan Wang, Jie Xu, Liu Yu, Maoyu Qin, Jianfeng Li, Sitian Liu, Weihan Zheng, Zeyu Li, Jun Ouyang, Yanbing Li, Guoxin Li, Ling Wang, Wenhua Huang, Yaobin Wu

**Affiliations:** 1 Guangdong Engineering Research Center for Translation of Medical 3D Printing Application, Guangdong Provincial Key Laboratory of Digital Medicine and Biomechanics, Department of Human Anatomy, School of Basic Medical Sciences, Southern Medical University, Guangzhou, 510515, China.; 2 Department of General Surgery, Nanfang Hospital, Southern Medical University, Guangzhou 510515, China.; 3 Biomaterials Research Center, School of Biomedical Engineering, Southern Medical University, Guangzhou, 510515, China.; 4 Department of Urology and Andrology, Ren Ji Hospital, School of Medicine, Shanghai Jiao Tong University, Shanghai 200001, China.

**Keywords:** injectable Janus hydrogel, photocrosslinking, all-in-one, minimally invasive, gastric perforations.

## Abstract

**Background:** Surgical sutures for sealing gastric perforations (GP) are associated with severe inflammation and postoperative adhesions. Hydrogel bioadhesives offer a potential alternative for sutureless repair of GP; however, their application in minimally invasive surgery is limited due to their prefabricated patch-form, lacking in situ gelation capability. In this study, we emphasized an all-in-one minimally invasive strategy for sutureless repair of acute GP.

**Methods:** an injectable photocurable Janus hydrogel was synthesized, and their ability to seal GP was performed. A rat GP model was used to verify the wound healing and antiadhesion efficiency of hydrogels, and a rabbit GP model was used to verify their laparoscopic feasibility. A fresh human corpse GP model was further employed to verify the user-friendliness of a minimally invasive deliverable (MID) device. A minipig GP model was utilized to evaluate the all-in-one minimally invasive strategy for the treatment of acute GP.

**Results:** Such injectable Janus hydrogel exhibited asymmetric adhesiveness, where the inner-facing side of the hydrogel displays strong sealing and wound healing abilities for GP, while the outward-facing side prevents postoperative adhesion formation. We further developed a minimally invasive deliverable (MID) device integrating hydrogel-delivery parts and photocrosslinking-gelation parts in a laparoscope system. The precise delivery and rapid fluid-tight sealing process of the injectable Janus hydrogel using the MID device for in situ GP repair were demonstrated in a simulated clinical scenario. The *in vivo* effectiveness of GP sutureless repair was successfully validated in porcine models, with further exploration of the underlying mechanism.

**Conclusions:** Our findings reveal that the injectable Janus hydrogel offers an all-in-one strategy for sutureless GP repair and concurrent prevention of postoperative adhesion formation by incorporating the MID device in minimally invasive surgery, presenting the significant potential to reduce patient surgical complications.

## Introduction

**Figure 1:**
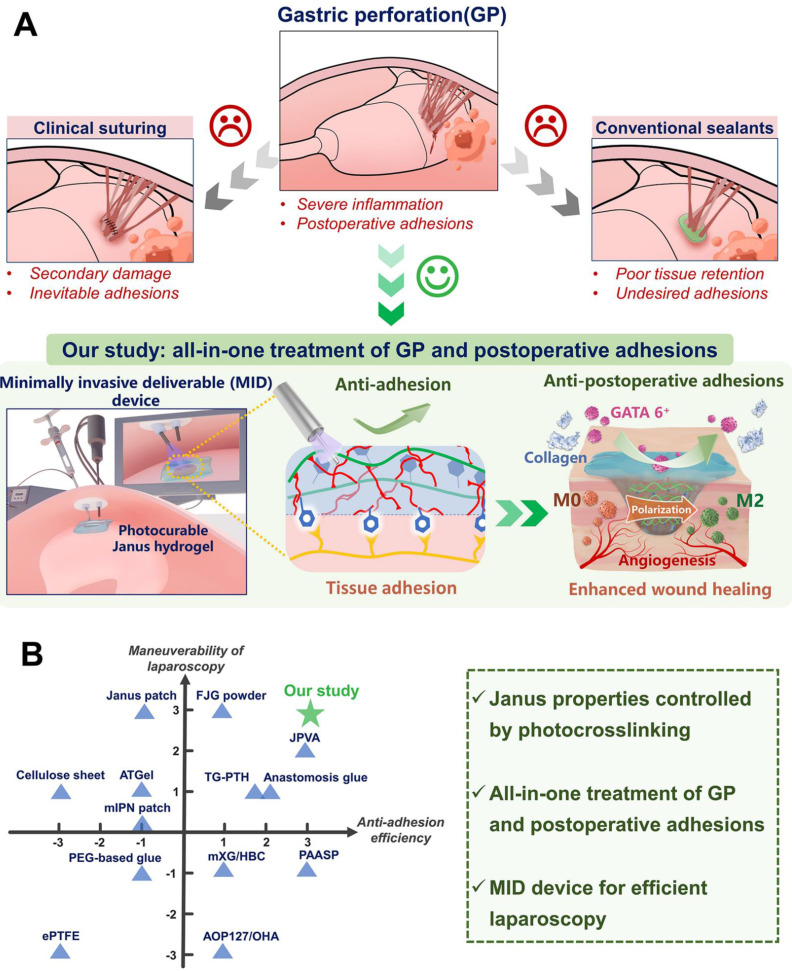
** Illustration of the all-in-one sutureless repair strategy integrating Janus HAD hydrogels with a minimally invasive deliverable (MID) device for GP repair. (A)** Schematic diagram of the complications of gastric perforations after surgery. Surgical suturing and conventional double-sided adhesion sealants and bioadhesives were applied to treat gastric perforations. Nevertheless, these strategies could not prevent postsurgical adhesions or inflammation. In contrast, our all-in-one sutureless repair strategy consists of Janus HAD hydrogels and the MID device for GP repair. **(B)** Comparison of the comprehensive properties of commercial sealants, reported bioadhesives, and our all-in-one strategy. The Ashby plot shows the relationship between the laparoscopic feasibility and efficiency of anti-adhesions.

Gastric perforations (GP) are a serious and potentially life-threatening condition that can be caused by a severe peptic ulcer, gastric cancer rupture, and surgical procedures 1. Leakage of intragastric contents mostly leads to a range of complications including postoperative adhesions, septic shock, severe pain, and even sepsis, with mortality rates increasing by more than 30% 2, 3. Surgical suture sealing is routinely used for GP rescue in clinical settings 4-6, while there remain limitations such as secondary tissue damage caused by needle piercing and the unavoidable development of postoperative adhesions 7-9 (Figure 1A). On the other hand, existing medical glues such as cyanoacrylate-based adhesives exhibit low biocompatibility, and difficult handling, resulting in poor integration with stomach tissues 10. Additionally, fibrin glues were also commonly used as clinical sealants in surgery, while their applications were constrained by poor adhesion strength, short degradation time, and high cost 11. In contrast, various advanced hydrogel-based bioadhesives have emerged as the most promising alternative for the sutureless repair of GP in recent years 12-14.

Among different hydrogel biomaterials for GP repair (Table S1), mussel-inspired hydrogels have been regarded as a type of synthetic bioadhesive that is well-suited for sutureless wound sealing, especially in emergency clinical settings. They possess superior tissue retention and excellent adhesive properties, enabling them to bond targeting interfaces effectively 15, 16. However, most of the present mussel-inspired hydrogel bioadhesives were designed for double-sided adhesion 17, 18, which presents a significant clinical problem due to indiscriminate adhesion on both faces and the resultant abdominal adhesions, severely limiting their potential for repairing internal organs, particularly in abdominal surgery 19. In light of these shortcomings, Janus hydrogels with single-sided adhesiveness have emerged as a potential solution to address the limitations of double-sided adhesives. For instance, a Janus poly (N-acryloyl 2-glycine) hydrogels patch was reported recently for repairing the stomach by bonding the perforated stomach surface *via* multiple hydrogen bonding interactions on one side, while simultaneously acting as a physical barrier on the other side to prevent postoperative adhesion 20. However, such pre-made patch applied for minimally invasive manipulation was difficult 21, due to their lack of ability to be administered *via* injection. In contrast, our recent study presents an injectable photocurable catechol-grafted hydrogel (HAD) grafted with catechol onto hyaluronic acid, capitalizing on its anti-adhesion clinical efficacy, and featuring asymmetric adhesive capabilities, thus rendering it suitable for anti-postoperative adhesion applications 16. The HAD hydrogel acted as a wet adhesive on the injured area, while remaining nonadherent on its outward-facing side after *in situ* gelation *via* photocrosslinking. This capability overcomes the drawbacks of the present patch-formed Janus barriers that needed manual press to realize tissue-adhesion 16. However, in more complex gastric perforation models, it is necessary not only to address postoperative adhesions but also to accurately deliver the hydrogel to the moist and dynamic gastric perforation site. Currently, there are no strategies available for surgeons to achieve precise and rapid delivery of the hydrogel in such scenarios. Unlike simply daubing hydrogel for anti-postoperative adhesion, precise delivery of the hydrogel bioadhesives to the SP area and prevention of leakage of gastrointestinal defects were both necessary for successful tissue sealing. Furthermore, gastric tissue with small-scale perforations can effectively expedite the healing process of the gastric mucosa with the aid of these injectable bioadhesives, thereby providing spontaneous acceleration to the overall gastric restoration 22. However, larger gastric perforations in larger animals are often challenged by leakage of gastric contents and peritonitis. Although Liu et al. recently reported an acid-tolerant and robust adhesion hydrogel for efficiently repairing rat gastric perforations 1, more *in vivo* work on larger animals still needs to be investigated to further demonstrate its potential for clinical GP repair application. Additionally, prolonged exposure to aqueous environments can cause a significant decline in the mechanical properties of injectable hydrogels, owing to their hydrogel network swelling behavior, which in turn leads to a loss of adhesion robustness. Recently, an injectable hydrogel composed of supramolecular assembled ABA triblock copolymers was applied for GP treatment 23. Nevertheless, when the intragastric pressure reaches a pathological level, the injectable hydrogel may rupture due to its decreased mechanical properties. In a brief summary, it remains an on-going challenge to develop an injectable hydrogel barrier for minimally invasive surgery, which would achieve robust sutureless GP repair and concurrent anti-postoperative adhesion abilities.

Herein, we present an injectable Janus hydrogel as an all-in-one treatment strategy for minimally invasive repairing GP and preventing postoperative adhesions. Our synthetic HAD hydrogel features asymmetric-adhesive properties controlled by the photocrosslinking process, where the inner-facing side of the hydrogel that interacts with stomach tissue exhibits fluid-tight sealing, while the outward-facing side displays nonadhesiveness. Furthermore, its interpenetrating hydrogel network was able to offer extraordinary long-term adhesion robustness and withstand intragastric bursting pressure in dynamic physiological environments. For laparoscopic manipulation, we further developed a minimally invasive deliverable (MID) device integrating hydrogel-delivery parts and photocrosslinking-gelation parts in a laparoscope system, which facilitated precise delivery and rapid fluid-tight sealing of the injectable Janus hydrogel during the laparoscopy (Figure 1A). Moreover,* the in vivo* effectiveness of GP sutureless repair and prevention of postsurgical adhesions was validated and the underlying mechanism was further explored in porcine models. We believe that integrating HAD hydrogel and the MID device would be a promising all-in-one strategy for the sutureless repair of gastric perforation, addressing the key limitations of hydrogel-based sealants or dressings. This strategy makes it easy for adequately trained surgical personnel to repair acute gastric perorations and effectively addresses the need for efficient surgical repair, thus serving as a reference for other biomedical material platforms.

## Materials and methods

### Materials

Hyaluronic acid sodium salt (HA, Mw: 1.0 × 10^6^ Da) was purchased from Shandong Huaxi Biological Technology. Ethyl(dimethylaminopropyl) carbodiimide (EDC), N-hydroxysuccinimide (NHS), 2-aminoethyl methacrylate hydrochloride (AEMA), dopamine hydrochloride and lithium phenyl-2,4,6-trimethylbenzoylphosphinate (LAP) were purchased from Sigma-Aldrich (Shanghai, China). TaqMan™ Reverse transcription Kit, SYBR Green PCR Master Mix, ELISA kit, and the other biological reagents above were purchased from J&L Biologica (Shanghai, China).

### Synthesis and characterization of HAD polymer

In brief, hyaluronic acid (1 g) was dissolved in 100 mL of deionized water at a 1% w/v concentration. EDC (4 mmol), NHS (4 mmol), and AEMA were gradually added and stirred in the solution for 30 to 40 minutes. Afterward, dopamine hydrochloride was included in the mixture for 24 hours. All solutions were shielded under nitrogen and stored in darkness at room temperature to prevent oxidation. The pH was monitored and adjusted to a range of 5 to 6 using 0.1 N hydrochloric acid and sodium hydroxide. Following the reaction, excess monomers were removed by dialysis (MWCO = 8000~12000 Da) for 72 hours under a pH of 5 and then lyophilized. The resulting lyophilized foam was stored in a sealed container and protected from light until use 16. The chemical structures of these HAD polymers were determined by nuclear magnetic resonance (^1^H NMR) and Fourier transform infrared (FT-IR). The ^1^H NMR spectra were recorded on a Bruker (AVANCEⅢ HD) 400 MHz spectrometer, using D2O as the solvent. The FT-IR spectra (NICOLET 6700, Thermo) were recorded in the range of 1500-3500 cm^-1^. The 0.5 wt% HAD hydrogel precursor solution was prepared, and the UV-vis spectra of the HAD precursor solution were recorded with a UV-vis spectrophotometer (Thermo Electron Corporation, USA) ranging from 200 to 650 nm. After freeze-drying, the cross-section and surface morphologies of the hydrogel samples were observed by a field emission scanning electron microscope (SEM, N7000, Hitachi).

### HAD hydrogels for sealing gastric perforations under intra-gastric pressure

Rat treated with surgical suturing and HAD hydrogels were sacrificed and their stomachs were collected. PBS was injected into the sealed rat stomach to evaluate fluid-tight sealing of the defects at the rate of 2 mL min^-1^ with a syringe pump. To measure burst pressure, a biological signal acquisition and analysis system (BL-420N, Chengdu) was used to monitor the applied pressure and record the real-time pressure curve 4. To quantitatively test the maximum adhesion and mechanical properties of HAD to withstand the sealing of gastric perforation, a testing setup was modified to examine the burst pressure (modified ASTM F2392-04 4). The testing setup consisted of an acrylic fixture, a syringe pump, and a pressure gauge. A bursting pressure assay was carried out to measure the adhesion strength of hydrogels according to the previously reported method 24. Moreover, after the burst test, the gastric tissues treated with HAD hydrogels were immersed in PBS for 24 hours to examine the wet-adhesiveness and fluid-tight sealing of the fully swollen HAD hydrogels. All experiments were repeated three times. Values were presented as mean ± standard deviation. Lap-shear test for the sol-adhesive" process and "gel-nonadhesive" process was detailed in our previous study 16. Briefly, the “gel-nonadhesive” process was conducted as follows. First, 300 μL of the HAD precursor with 0.1 wt% LAP was smeared evenly on the fresh porcine skin substrate. The HAD precursor was then crosslinked under UV irradiation (365 nm) for 60 seconds. After the HAD hydrogel was formed, the inner surface of the hydrogel was adherent to the porcine skin, while the outer surface of the hydrogel was nonadherent. Subsequently, another fresh porcine skin was put onto the outward side surface of HAD hydrogel, and the adhesion properties were tested and recorded using the Materials Test system (MTS Criterion 43, MTS Criterion) equipped with a 50N load cell at a rate of 10 mm/min.

### Assessing the effect of HAD hydrogels on GP repairing, wound healing, and anti-adhesion in rat models

Male SD rats (220-250 g) were divided into three groups of 6. Animals fasted for 24 h, and were then anesthetized with 1% sodium pentobarbital (40 mg/kg), and received a 4cm midline incision. A 5mm gastric defect was created and repaired with HAD hydrogels or sutures (8-0 Prolene) (n = 4/group). 200 μL of the HAD precursor was injected into the hole before covering it with HAD solution (3w/t%). UV light (365nm) crosslinked the HAD precursors with the gastric perforation (15-20s). Animals were euthanized on day 7 and 14 post-surgery for fluid-tight sealing confirmation and histological/immunofluorescence analyses of collected stomach samples. Gross observation and adhesion score refer to our previous study and Table S2 (Supporting Information) 16.

### Assessing the laparoscopic feasibility of HAD Hydrogels for GP repair in rabbit models

New Zealand white rabbits underwent laparoscopic surgery after fasting for 24 h. Anesthetized with 3% sodium pentobarbital, rabbits were fixed in a supine position with three channels established on the abdominal wall. The first channel (5mm) established pneumoperitoneum, the second channel (12mm) allowed HAD precursor injection and laparoscopic instruments operation, and the third channel (10mm) set the UV light tube for crosslinking HAD hydrogels. After preparing the artificial wound, sterilized HAD precursors were injected and immediately crosslinked in situ for 10 seconds with UV light (30W/cm2, OmniCure, SERIES 2000, USA). The entire surgical procedure took approximately 5 minutes. Rabbits treated with surgical sutures were considered the Suture group, and all rabbits were observed and histologically examined at predetermined times after sacrifice.

### MID device fabrication and application in simulated clinical scenarios

Our multipurpose integrated and minimally invasive deliverable (MID) device consists of two parts including an injection part and a UV part, respectively. The injection part comprises five optical fibers parallelly wrapped to a 20-gauge needle housed within an 8-gauge needle, using a simple fiber cleaving and splicing fabrication protocol for UV transmission. The UV part transmits 365 nm UV light from an external light source (30 W/cm^2^, OmniCure, SERIES 2000, USA) to the body cavity *via* the optic fiber bundles wrapped around the injection needle. The Department of Human Anatomy at Southern Medical University prepared a fresh human body corpse for our study, which was approved by both the Southern Medical University Ethics Committee and the Medical Ethics Committee of Nanfang Hospital (Ethical authorization number: NFEC-2022-381). Two minimally invasive defects were made on the abdomen to accommodate the MID device and the camera port (MedlabTM). The surgeons from Nanfang Hospital precisely delivered 500 μL of ICG-labeled HAD precursor to cover the perforation site and induced in situ gelation of HAD hydrogels *via* UV photocrosslinking using the MID device.

### qRT-PCR and Western blotting analysis

This study measured mRNA levels of TGF-β1, TNF-α, and IL-6 in the local adhesion tissues of rats. Three rats in each group were euthanized on Day 7 and Day 14, and the adhesion tissue was collected. Total RNA was extracted, cDNA was synthesized, and quantitative real-time PCR was performed using SYBR Green Master Mix. Relative gene expression changes were calculated using the 2-ΔΔCt method. The endogenous control was GAPDH. The primer sequences used were shown in Table S3. Total protein extracted using RIPA buffer, quantified by BCA kit, and adjusted by loading buffer. Separated in 10% acrylamide gel & transferred to PVDF membranes. Blocked with 5% skim milk powder for 1 h, incubated with primary antibodies at 4 °C overnight, then with secondary antibodies conjugated to horseradish peroxide diluted in blocking buffer for 2 hours. Protein bands detected by ECL luminescence kit.

### Sutureless GP repair and anti-adhesion using the integration of HAD hydrogel and the MID device in pig models

All animal experiments were approved by the Ethics Committee of Southern Medical University. Three adult female minipigs (35-40 kg) underwent gastric perforation induction* via* artificial incisions while under sedation with diazepam (0.2 mg/kg) and anesthesia with inhaled isoflurane (1.5-2.5%). The MID device was used to create 15mm-diameter minimally invasive abdominal defects, and the stomach was accessed and perforated using a 5mm-diameter needle. Animals were randomized to receive HAD hydrogel (500 μL), surgical suturing (12 cm^2^), or no treatment. HAD precursors were delivered to the site via the MID device and photocrosslinked for 5-10 s to induce ultrafast in situ gelation. Electrocardiogram and vital signs were monitored throughout the procedure and post-operative pain control. Surviving animals were euthanized after two weeks to assess perforation recovery.

### Histology and inflammation grading

During the first and second weeks after surgery, the animal's stomach was harvested. To remove excess blood, the stomach was flushed with PBS and then cut horizontally into three 5 mm thick slices. These slices were fixed with 4% paraformaldehyde for 4h before being dehydrated overnight with 30% sucrose for cryostat sectioning. For analysis, all gastric perforation samples were sectioned at a thickness of 5μm and subjected to hematoxylin and eosin (H&E), Masson's trichrome staining, and immunostaining. The architecture of glands was graded as follows: grade 0, almost normal glandular structure; grade 1, gastric glands arranged sparsely with some slightly dilated; grade 2, morphologic changes between 1 and 3; grade 3, gastric glands are dilated markedly with distorted architecture and epithelial cells are poorly differentiated 25.

### Statistical analysis

The results of all *in vivo* tests were presented as mean ± standard deviation and analyzed using the Shapiro-Wilk test followed by Student's t-test. A significance level of *P* < 0.05 was considered statistically significant. GraphPad Prism software (GraphPad Software Inc.) was used for data analysis.

## Results

### Injectable Janus HAD hydrogel for sutureless sealing of GP *in vitro*

**Figure 2:**
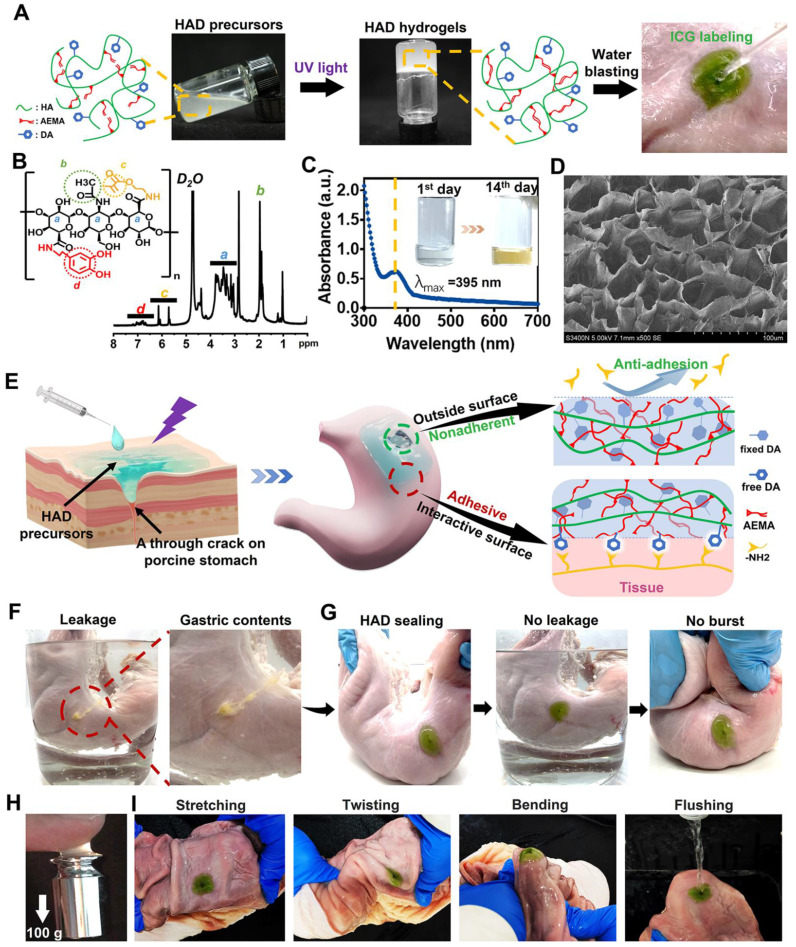
** HAD hydrogel for sutureless and robust sealing of GP *in vitro*. (A)** HAD hydrogel was formed after photocrosslinking. **(B)**^1^HNMR spectra of HAD polymer, where “a” represents the protons in the ring structures of HA (δ = 4.0-3.0 ppm), “b” represents the C(=O)CH_3_ in HA (δ = 2.1 ppm), “c” represents the C=C in AEMA group (δ = 5.68 and 6.13 ppm), and “d” represents the protons in the catechol ring in DA group (δ = 6.5-7.2 ppm). **(C)** UV-vis spectra of HAD hydrogel solutions after oxidation at 37 °C. Quinone peak: λ max (ε) = 395 nm, **(D)** SEM micrographs confirmed the porous microarchitecture of the lyophilized HAD hydrogels. **(E)** Schematic diagram of the Janus HAD hydrogel for robust and efficient sealing of stomach tissues: The inner-side surface of HAD hydrogels could form robust adhesion on the stomach surface due to the Michael-type reaction, while the outward-side face of HAD was nonadherent due to the restriction of free DA groups after photocrosslinking. **(F)** The clear leakage consisting of gastric contents was bursting from the crack of the porcine stomach. **(G)** HAD hydrogels sealed the 5mm-diameter gastric crack, preventing leakage and withstanding the exaggerated extrusion. **(H)** After photocrosslinking, the HAD hydrogel stuck to fingers, withstanding a weight of 100 g. **(I)** HAD hydrogel maintained stable adhesion and integrity on the stomach tissue crack site after stretching, twisting, bending, and water flushing.

The injectable photocurable asymmetric-adhesive hydrogel (HAD) was synthesized by grafting methacrylate and catechol groups on the hyaluronic acid polymer chain (Figure 2A-B), whereas the detailed synthesis descriptions were confirmed in the Supplementary data (Figure S1A-C). The analysis of zeta potential revealed that HA solutions displayed a zeta potential of approximately -57.3 ± 0.7 mV at pH 7.4. In contrast, the zeta potentials of the HAD precursor solutions ranged from -42.3 ± 1.1 to -38.3 ± 1.3 mV, indicating an increase in positive charge due to the presence of the DA and AEMA groups (Figure S1D). The injectability of hydrogels for laparoscopic applications is greatly influenced by their viscosity properties. At 37 °C, the viscosity of the HAD precursor solution exhibited an exponential decrease as the shear rate was increased (Figure S1E). This behavior indicated that the HAD precursor solution possesses favorable shear thinning characteristics, making it suitable for easy administration through injection. The instant and ultrafast *in situ* gelation (< 5 s) of HAD hydrogels was achieved under UV irradiation using LAP as the photoinitiator, as was evidenced by the capability to withstand the running deionized water (Figure 2A). UV-vis spectroscopic analysis indicated that the quinone peak of the HAD precursors did not appear after oxidation for 14 days, indicating the slow oxidation of catechol groups in HAD (Figure 2C), which is important as it ensures the continuous adhesiveness of catechol-based hydrogels 25. SEM micrographs confirmed the porous microarchitecture of the lyophilized HAD hydrogel as shown in Figure 2D. Once the HAD hydrogel formed *in situ* gelation on the porcine stomach, the strikingly distinct adhesive and nonadhesive properties on two-side faces of the HAD hydrogel were presented (Figure 2E). The Janus HAD hydrogel formed *in situ* adhered well to the wet surface of the porcine stomach due to the Michael-type reaction between the free-floating DA groups on HAD and the amino or thiol groups on stomach tissues 15. In contrast, the outside surface of the Janus HAD hydrogel failed to adhere to the porcine tissues, demonstrating the anti-adhesion behavior, due to the establishment of the AEMA network after photocrosslinking, which limited the free movement of DA groups in HAD and prevented them from interacting with the stomach surface nucleophiles. Thus, the outside surface of the crosslinked HAD was also nonadherent to internal organs. The asymmetric adhesiveness of the Janus HAD hydrogel was further verified on the stabbed porcine stomach. A deep wound to the stomach caused gastric contents leakage in the ambient liquid environment (Figure 2F). However, the robust sealing of HAD hydrogel prevented the gastric leakage from the stomach crack from contaminating the surrounding clear liquid (Figure 2G). Moreover, the strong adhesion formed between the HAD hydrogel and the porcine stomach could also withstand the exaggerated expansion and extrusion (Figure 2G). In addition, under UV irradiation, a crosslinking network was formed *in situ* to achieve strong tissue adhesion, allowing the HAD hydrogel to adhere to tissues and withstand a weight of 100 g (Figure 2H). Even being treated by repeated distortion, bending, stretching, and water flushing, no obvious detachment of HAD hydrogels adherent to the inner or outer stomach wall was observed (Figure 2I, Figure S2-S3A). However, the outside surface of the HAD hydrogel exhibited nonadhesiveness and failed to adhere to the paper napkin due to the restriction of the free-floating DA groups in HAD (Figure S3B). As seen in Figure S3C-D, we observed the interaction between Alexa Fluor 594-conjugated fibrinogen solution (depicted in red) and thrombin with the fluorescein isothiocyanate (FITC)-labeled HAD precursor (shown in green) on the surface. The subsequent photocrosslinking process led to the formation of the hydrogel, referred to as the "sol-adhesive" process. Fluorescence imaging confirmed that the fibrin adhered to the HAD hydrogel's surface due to the Michael-type reaction between the catechol groups on HAD and the amino or thiol groups on fibrin (Figure S3C). For assessing the nonadhesive performance of crosslinked HAD formulations, we initially photocrosslinked the HAD precursor to form the hydrogel. Then, a fibrinogen solution and thrombin were applied to the surface of the crosslinked HAD gel, termed the "gel-nonadhesive" process. After 120 min incubation, the hydrogel's surface was rinsed with deionized water. Fluorescence microscope images revealed the absence of fibrinogen deposition (in red) on the green HAD, underscoring that the fibrin did not adhere to the crosslinked HAD surface (Figure S3D). This outcome stemmed from the establishment of the AEMA network, which restricted the movement of catechol groups within HAD following photocrosslinking.

### HAD hydrogel replaced sutures withstanding supraphysiological burst pressure to prevent gastric leakage

To further quantitatively evaluate the asymmetric adhesion properties of HAD hydrogel, we designed two different lap-shear tests to investigate the adhesive strength between the hydrogels and porcine skin following the "sol-adhesive" and "gel-nonadhesive" processes, respectively. For the "sol-adhesive" process, the HAD hydrogel precursors were placed between two pieces of fresh porcine stomach tissues, after which UV light was applied for photocrosslinking.

**Figure 3:**
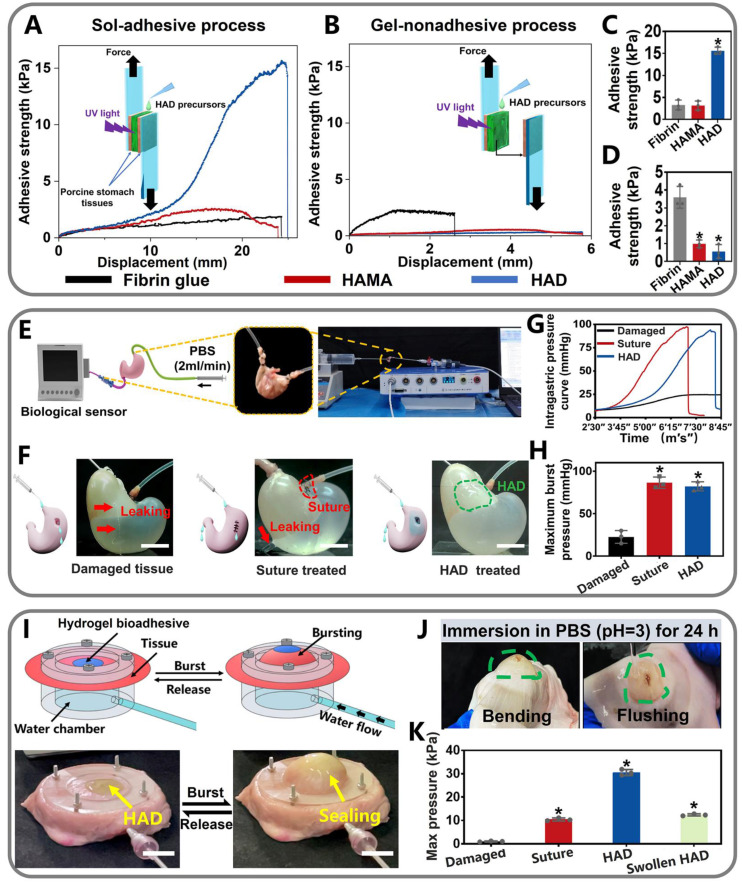
** Quantitative measurement of the asymmetric adhesiveness and sutureless GP sealing of HAD hydrogels. (A-B)** Schematics showing the lap-shear test procedure following the "sol-adhesive" **(A)** and "gel-nonadhesive" **(B)** processes. The adhesive strength-displacement curves of the “sol-adhesive” and “gel-nonadhesive” processes were recorded. **(C-D)** Adhesive strength between the hydrogels and stomach tissues following the "sol-adhesive" and "gel-nonadhesive" processes. *p< 0.05 and **p< 0.01 compared with fibrin glue group. **(E-F)** Rat stomachs with 5 mm diameter defects were treated with surgical suture and HAD hydrogel, respectively, followed by recording the real-time bursting pressure with a biomechanical sensor (BL-420N, Chengdu). Scale bars = 1 cm. **(G)**, The real-time intragastric pressure curve of each group. **(H)**, The maximum bursting pressure of the untreated, suture, and HAD group (*P < 0.05 compared with the model group, n = 3 independent samples). **(I)** Schematic diagram of the bursting press test and the bursting moment of each group. Bursting pressure test of *ex vivo* porcine stomach with a 5 mm diameter defect sealed by surgical sutures and HAD hydrogels, respectively. Black arrow represented the sealing achieved by HAD hydrogels. Scale bars = 1 cm. **(J)** HAD hydrogels still robustly adhered to the *ex vivo* porcine perforation when imposed with stretching and torsion after immersion in PBS (pH =3) for 24h. **(K)** Results of maximum bursting pressure of gastric defect sealed by surgical sutures, HAD hydrogels, and the swollen HAD hydrogels, respectively. The gastric defect without treatment was considered as the control group (*P < 0.05 compared with the model group, n = 3 independent samples).

Adhesion strength was recorded after stable adhesion was formed between the hydrogel and the two pieces of the porcine stomach (Figure 3A). In contrast, for the "gel-nonadhesive" process, the HAD hydrogel precursors were firstly completely crosslinked on one piece of the fresh porcine stomach. The second piece of the fresh porcine stomach was then attached to the surface of the crosslinked HAD hydrogel (Figure 3B). The commercially available fibrin glue (Greenplast^®^) and HAMA hydrogel with no grafting DA groups were considered as the control groups. Under the sol-adhesive process, HAD hydrogels possessed significantly higher adhesive strength (15.98 ± 1.12 kPa) than HAMA hydrogel (3.12 ± 0.89 kPa) (p < 0.01) (Figure 3C). HAD hydrogel also exhibited greater adhesion strength than commercial fibrin glue (about 2.31 kPa ± 0.19 kPa). However, under the gel-nonadhesive process, the adhesion strength of both the HAD and HAMA hydrogels decreased to approximately 0 kPa (p < 0.01) (Figure 3D). The lap-shear test results demonstrated that the HAD hydrogel achieved asymmetric adhesiveness due to the control of photocrosslinking and catechol group (DA) grafting.

Further quantitative experiments were performed to evaluate the sutureless sealing ability of HAD hydrogels. Rat stomachs were constantly filled with water and punctured to form artificial defects (diameter=3 mm), treated by HAD precursors or surgical sutures, respectively (Figure 3E-F). For the HAD-treated group, the sealing formed by HAD hydrogels exhibited an average burst pressure of >11.19 kPa (84 mmHg), while the suture-treated rat stomach exhibited an average burst pressure of > 11.33 kPa (85 mmHg) (Figure 3G-H and Video V1). We continued to use the porcine stomach to perform fluid-bursting pressure tests(Figure 3I and Figure S4) 4. Due to the rapid and robust adhesion formed by HAD hydrogels, the actual burst pressure of HAD was 30.1 ± 1.3 kPa before swelling (Video V2), which was significantly higher than the normal physiological intragastric pressure of human and the surgical sutures treatment (average burst pressure >10.2 ± 1.0 kPa) (Figure 3I-K) 26, 27. Moreover, the HAD hydrogel swollen in phosphate-buffered saline (PBS) for 24 h also maintained good adhesion on the stomach surface with a maximum bursting pressure of 12.2 ± 1.1 kPa (Figure 3J-K and Figure S5). Hence, HAD would still adhere to the stomach tissue even after swelling in the peritoneal fluid due to the strong interactions between the catechol groups of HAD and the natural nucleophiles (amido bonds, thiols, and amines) on the stomach tissue surface. Overall, the adhesiveness of HAD hydrogels exhibited the potential to replace sutures. The robust adhesion and fluid-tight sealing were formed at the defects by the ultrafast gelation of HAD hydrogel only within 1~3 s *via* photocrosslinking, which was significantly shorter than the time of suturing (at least > 3 min).

### HAD hydrogel for GP repair and simultaneous anti-postoperative adhesion in rat models

**Figure 4:**
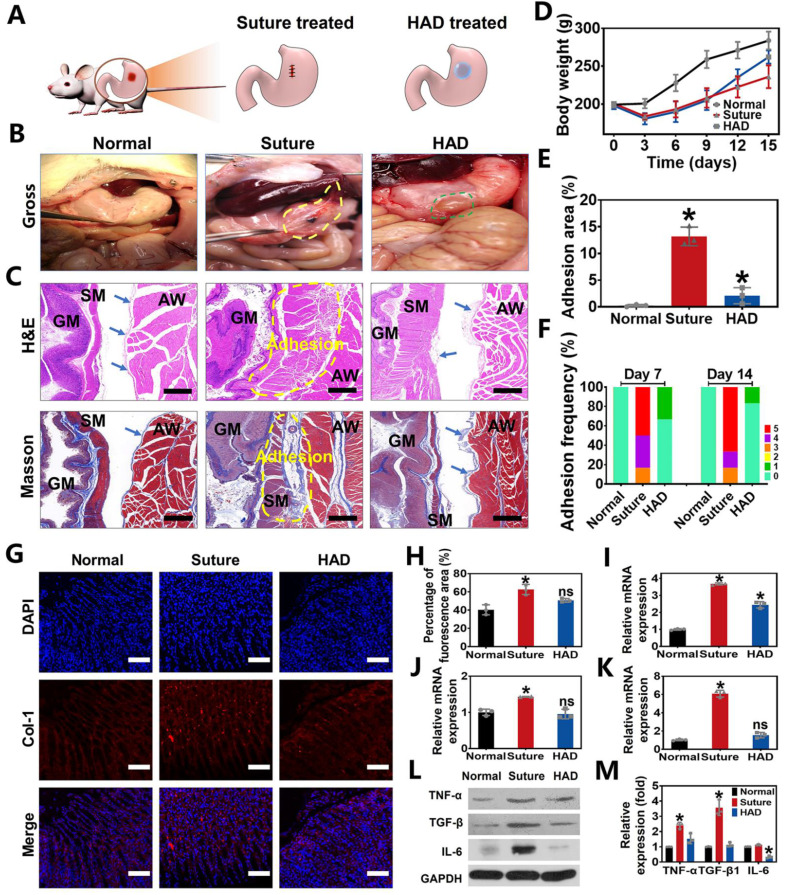
** Perforation wound healing and anti-postoperative adhesion efficiency of HAD hydrogel after abdominal surgery in rat GP models. (A)** Schematic diagram of HAD hydrogel or suture treatments in rat GP model following the open laparotomy process. **(B)** Macroscopic images of rat stomach perforation sites of each group at 14 days after surgery. Yellow circles represented the postoperative abdominal adhesions. Green circles represented the residues of HAD hydrogel. **(C)** The representative images of H&E staining and Masson staining of locally treated tissues in each group on day 14. GM, gastric mucosa; SM, smooth muscle; AW, abdominal wall. The blue arrows pointed to mesothelial cells. The adhesion sites were marked with dotted yellow lines. Scale bars = 200 µm. **(D)** The average weight of rats in each group after surgery. **(E)** Percentage of adhesion area in each group, *p < 0.05 and **p < 0.01 compared with the normal group. **(F)** Adhesion scores of each group on day 7 and day 14 after surgery (n = 6). **(G)** Immunofluorescence images of tissue sections in each group that were stained with DAPI (blue) and COL-1 (red) on day 7 after surgery. Scale bars = 100 µm. **(H)** Percentage of COL-1 fluorescence area of local treated tissues in each group on 14 days. **(I-K)** Related mRNA expressions of TNF-α, TGF-β1, and IL-6 in each group on day 14 after surgery. **(L)** Western Blot assay results in each group on day 14 after surgery. **(M)** All Western Blot bands of experiment groups were normalized to glyceraldehyde 3-phosphate dehydrogenase (GAPDH) bands (*P < 0.05 compared with the model group, n = 3 independent samples).

To evaluate the *in vivo* efficacy of HAD hydrogels in treating gastric defects and simultaneously prevent postoperative adhesion, we created 5mm-diamater artificial defects on the stomach wall in rat GP models, which were subsequently treated with HAD hydrogels and sutures, respectively (Figure 4A). As seen from the treatment by the open laparotomy, we found puncture-driven tissue damage could be caused by the surgical stitches (Figure S6). Worse yet, the process of suture fixation usually took at least 5 min, leading to imperceptible bleeding points and prolonging the operative time. In contrast, the application of HAD hydrogels achieved atraumatic and instant sealing of gastric contents and bleeding within 15 s without further surgical sewing. After surgical treatment for 7 days, the suture group showed severe fibrous adhesion connecting the suture knots with an average adhesion score of 4.33 ± 0.1 (Figure 4F and Figure S7). On day 14 after the operation, the adhesion tissues (an average score of about 4.5 ± 0.2) in the suture group were thicker than before, and the obvious fibrous tissues between the stomach and nearby organs such as liver and intestines (Figure 4B and F). In contrast, rats treated with HAD hydrogel showed a favorable efficiency of perforation repair and anti-postoperative adhesions, with an average adhesion score of 0.33 ± 0.1 and 0.17 ± 0.1 on day 7 and 14, respectively (Figure 4B and F). Notably, the inner side of the HAD hydrogel residues was found to be tightly adhered to perforation sites (Figure 4B and Figure S7), while the outer side was nonadherent, thus preventing leakages and adhesion formation effectively due to the asymmetric-adhesiveness of HAD hydrogels. In addition, the degradation of HAD hydrogels did not cause obvious symptoms of inflammation, such as exudation, condensation, or infiltrate in major organs during the 14 day-period of gross and pathological observation (Figure S8), which was in consistent with the results of *in vivo* degradation experiment (Figure S9), indicating a proper* in vivo* retention time (about 2 weeks) of HAD hydrogels. Besides, macroscopic examination of the harvested gastric tissues also revealed a significantly faster increase in postoperative body weight and smaller adhesion area in the HAD group both on 7 and 14 days (Figure 4D-E). In addition, the capability of HAD hydrogel for GP repair was further investigated by histology staining. For the suture group, mass inflammatory cell infiltration such as neutrophils and mononuclear macrophages was observed between the gastric wall and the abdominal in the suture group from the H&E staining, and large-scale collagen deposition (blue area) was fused with smooth muscles of the gastric wall and skeletal muscles of the abdominal wall as shown in the Masson staining images, leading to the serious peritoneum and destruction of gastric mucosa at suture tissues (Figure 4C and Figure S10). Contrastively, as shown in H&E and Masson staining results, it was observed that the HAD hydrogel group showed almost no inflammatory adhesion or collagen deposition on both days 7 and 14. Although some area of the mesothelial cell layer of the gastric wall was disorderly arranged on day 7, the structures such as gastric pits, mucosa layer, and smooth muscles were identified on day 14, indicating that HAD hydrogel was capable of blocking gastric perforations, promoting wound healing, and reducing abdominal adhesions in the complex and acidic *in vivo* environment. Moreover, the marker of type I Collagen (COL-1) (red fluorescence) in the local adhesion tissues of the suture group was stronger and more than that of the HAD gel group. The local tissues of HAD hydrogel-treated rats showed a comparable red fluorescence intensity to normal animals (Figure 4G-H). The relative mRNA expressions of TNF-α, TGF-β1, and IL-6 in the HAD, suture, and normal group were further detected by RT-PCR (Figure 4I-K and Figure S11). The surgical-treated animals showed the highest mRNA expression level of TNF-α, TGF-β1, and IL-6 either on day 7 or day 14 after surgery. Compared with the suture group, the HAD gel group exhibited lower expressions of adhesion-related inflammatory factors* in vivo*. In particular, expressions of TGF-β1 and IL-6 decreased to a level that was not significantly different from the normal group on day 14 (*p* > 0.5). Protein expression levels of related inflammatory factors were detected by Western Blot assay, which was consistent with the mRNA expression level. (Figure 4L-M and Figure S12). Results of the observational evaluation and pathological evaluation proved that the injectable HAD formulations not only promoted the healing of the mucosa layer and mesothelial cell layer of the stomach but also improved the prognosis of postoperative adhesion formation.

### Laparoscopic feasibility of HAD hydrogels for GP treatment in rabbit models

**Figure 5:**
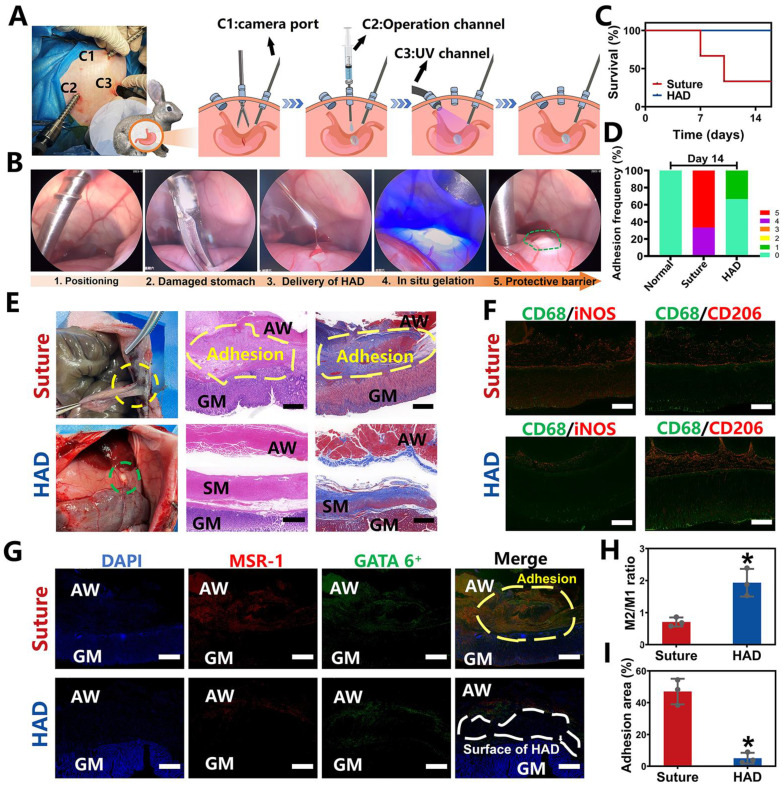
***Ex vivo* demonstrations of HAD hydrogels for minimally invasive delivery in rabbit GP models. (A)** Schematic illustration of the process by which HAD formulations were applied on stomach perforation sites created by resection, followed by UV crosslinking in rabbits through laparoscopic surgery. The establishment of three channels on the rabbit abdomen and the imaging system was prepared for the laparoscopy. Three channels included the laparoscopic camera port channel **(C1)**, operation channel **(C2)**, and UV light channel **(C3)**. **(B)** Laparoscopic imaging of the laparoscopic procedures of creating artificial wounds on the stomach wall by laparoscopic resection, the HAD hydrogels injection, and UV crosslinking. **(C)** Survival of rabbits after laparoscopic surgery and treatment with HAD formulations or saline solution within 14 days (n = 3). **(D)** Adhesion scores for each group at predetermined time points (n = 3) **(E)** Macroscopic images of the operated rabbit cecum on day 14 after laparoscopy. SM, smooth muscle; AW, abdominal wall; GM, gastric mucosa. Scale bars = 200 µm. **(F)** Immunofluorescence images of marrow-derived macrophages in perforation tissues after treatment stained with inducible nitric oxide synthase (iNOS, red), CD206 (red), and CD68 (green). Scale bars = 50 µm. **(G)** Immunofluorescence images of tissue sections including GATA6^+^ macrophages (green), MSR-1 (red), and DAPI (blue), respectively. SM, smooth muscle; AW, abdominal wall; GM, gastric mucosa. Scale bars = 500 µm. **(H)** Ratio of M2 to M1 analysis of the suture and HAD groups. **(I)** Percentage of adhesion area in each group (*P < 0.05 compared with the model group, n = 3 independent samples).

To further explore the clinical translational potential of HAD formulation in minimally invasive surgeries, the laparoscopic feasibility of HAD hydrogels for GP treatment in rabbit models was investigated. As shown in Figure 5A, after pneumoperitoneum was established (channel 1, C1), the HAD precursor was applied topically to the site of gastric perforation with a long needle passing through the laparoscopic trocar (channel 2, C2). This was followed immediately by photocrosslinking (channel 3, C3) with an external UV light spot curing system (30 W/cm^2^) to induce ultrafast gelation* in situ* to form an instant protective barrier. Compared with suture treatment (suture group), the entire operation of sealing the gastric perforation with HAD hydrogels was less time-consuming and more convenient without meticulous hemostasis (Figure 5B and Video V3). All the rabbits in the HAD group survived after laparoscopic surgery, while the suture group showed a mortality rate only with only 33.3% (Figure 5C). From the macroscopic examination on day 14 after laparoscopy, the HAD residues remained robustly adhered to the perforation with no appearance of tissue edema or inflammatory response, with an average adhesion score of 0.3 ± 0.1 (Figure 5D-E). In contrast, a thick fibrotic adhesion was observed in the suture group, with vascularized tissues connecting the stomach and abdominal wall with an average adhesion score of 4.7 ± 0.1 (Figure 5D-E). H&E and Masson staining results further revealed that the local stress created by penetration during suturing resulted in drastic deformation of the mucosal layer and contraction of the full-layer stomach, while typical three-layered structures of the gastric walls around the perforation were observed in HAD group after 14 days (Figure 5E). In addition, as seen in Figure 5F-H, the immunofluorescence staining with CD68^+^ (macrophage marker), iNOS^+^ (M1 marker, proinflammatory phenotype), and CD206^+^ (M2 marker, anti-inflammatory phenotype) showed the dramatic enrichment of CD206^+^ macrophages during the degradation process of the HAD hydrogels. The investigation of the dynamics of marrow-derived macrophage polarization indicated that HAD bioadhesives were able to promote the transition from the M1 to M2 phase 28-30. Considerable aggregation of peritoneal GATA6^+^ macrophages (green) and MSR-1 (red) was found between the gastric perforation and the abdominal wall with adhesions connecting them in the suture group. In contrast, immunohistochemical staining in the HAD group showed that only a few GATA6^+^ macrophages remained without obvious adhesion formation between the peritoneum and the stomach (Figure 5G-I). These results were consistent with our previous study 16, demonstrating that HAD hydrogels captured free-floating GATA6^+^ cavity macrophages by neutralizing MSR-1 since the HAD formulation contained a polyanionic ligand. Consequently, the free-floating GATA6^+^ macrophages failed to recognize already captured macrophages, thus inhibiting the super-aggregation of GATA6^+^ macrophages around the gastric perforation. Overall, the application of the injectable and asymmetrically adhesive HAD hydrogels in laparoscopy showed that they are suitable for suture-free wound closure and for delivery during minimally invasive manipulation to form an instant protective barrier *in situ* with prominent wound healing and anti-adhesion effects.

### The rapid sealing ability of HAD hydrogel integrating the MID device in a simulated clinical scenario

**Figure 6:**
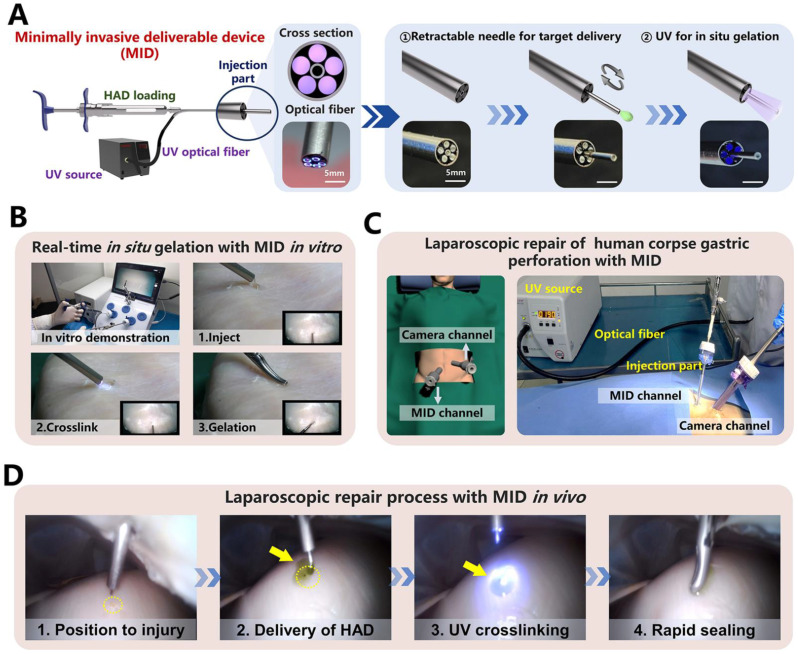
** The laparoscopic process of HAD hydrogel integrating the MID device in a simulated clinical scenario. (A)** Schematic diagram and fabrication details of the MID device, which consists of hydrogel-delivery parts and photocrosslinking-gelation parts in a laparoscope system. Five optical fibers were arranged in parallel along the inside of a retractable needle, thus transmitting external UV light to the body cavity to induce the gelation of HAD hydrogels. Steps for using the MID device in laparoscopy: The injection needle of the MID device passed through the sleeve for target delivery of HAD. The UV light was then transmitted via the MID device to induce the real-time *in situ* gelation of HAD hydrogels by surgeons to form instant barriers at perforations. **(B)** Simulation laparoscopy for sealing *in vitro* porcine defects through the MID device in a laparoscopic training box. **(C)** Schematic illustrations for the sutureless GP repair in a fresh human corpse. Two channels included the laparoscopic camera port channel and the operating channel for the MID device. Yellow circles represented perforation injuries. Yellow arrows represented the ICG-labeling HAD hydrogels. **(D)** Laparoscopic imaging of creating an artificial defect (diameter = 5mm) on the stomach of the fresh human corpse by laparoscopic resection, HAD hydrogel delivery, and UV crosslinking through the MID device.

Despite the operating process of HAD hydrogel during a minimally invasive surgery was successful, the separation of the injection and photocrosslinking steps would prolong the operation time with higher risks of trocar-related complications (Figure S13), thereby limiting the practical application for HAD hydrogels in acute clinical scenarios 31, 32. Therefore, to overcome the separated procedures of HAD delivery and UV crosslinking in laparoscopy, we designed a minimally invasive deliverable (abbreviated as MID) device that integrated the capacities of hydrogel delivery and real-time photocrosslinking with a single trocar channel. As illustrated schematically (Figure 6a), the key structure of the MID was a coaxial injection part, which consisted of the 20 and 8G needles as the inner and outer channels, respectively. For the cross-section view, it was clearly observed that five optical fibers arranged around the inner channel were applied for UV light t transmission, and the inner channel was used for HAD precursor delivery. Especially, the inner channel was designed as a retractable part, which would be beneficial for not only precisely delivering hydrogel on the target site but also for protecting the optical fibers out of the contamination of hydrogel precursor (Figure S14). The optimal size of the fibers was experimentally determined by studying the relationships among the fiber length, fiber diameter, and the power intensity of UV light (Figure S15). When the length of the fiber was decreased to 50 cm, the UV light intensity was kept at approximately 4 mW/cm^2^, which was the minimum UV power intensity to form the HAD hydrogels. To simulate the application of the MID device in a minimally invasive surgical setting, the sealing of an injured porcine stomach* in vitro* was performed inside a dark laparoscopy training chamber (Figure 6B and Video V4). To further verify the feasibility of surgeons using the MID in real scenarios, a fresh human corpse was used in a procedure that was approved by the Southern Medical University Ethics Committee and the Medical Ethics committee of Nanfang Hospital. Using this MID device, only two standard abdomen trocar channels were needed to perform the operation (Figure 6C and Figure S16-17). After creating artificial gastric defects in the human stomach, a proper amount of indocyanine green-labeled HAD precursors were precisely delivered to completely cover the target site, followed by UV photocrosslinking* via* the optical fibers of the MID device (Figure 6D). It was observed that both the amount of HAD precursor and the UV irradiation time could easily be controlled by one surgeon and all the operation processes could be successfully performed only in two trocar channels (One channel was for necessary imaging and the other was for MID device), thus saving the preparation and operation time for clinical emergency (Video V5). Even with mucus leaking from the stomach, the asymmetrically adhesive HAD hydrogels adhered tightly to the perforation, thereby providing a stable protective barrier to promote perforation healing and prevent indiscriminate adhesion to surgical instruments or other normal organs. It took only 5 minutes to deliver HAD hydrogels and form a complete cover and stable protective barrier at the irregular wounds in body fluid-rich environments. Laparoscopic delivery of the injectable and photocurable HAD hydrogel precursors via the user-friendly MID device served as an important simulation of clinical practice, and the method proved to be ideal for providing a rapid and robust protective barrier of gastric leakage and effectively promoting perforation healing after a minimally invasive procedure. These data suggested that integrating HAD hydrogel with the self-designed MID device would be a promising all-in-one strategy for the sutureless repair of gastric perforation.

### Laparoscopy performance following the all-in-one sutureless strategy for GP rescue and anti-postoperative adhesions in pigs

**Figure 7:**
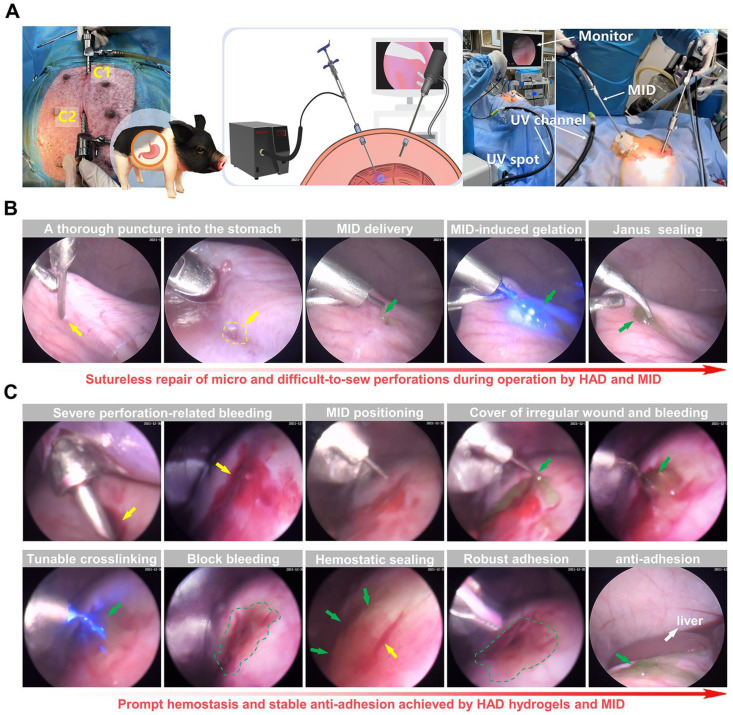
** Sutureless repair of acute gastric perforations in minipig models following the all-in-one strategy. (A)** Schematic illustration of the laparoscopic process of the all-in-one strategy for *in vivo* GP repair in porcine models. Establishment of two channels on the minipig abdomen, including the camera port channel **(C1)**, and operation channel **(C2)**. **(B)** Laparoscopic demonstration of rapidly sealing the micro and difficult-to-sew intraoperative perforations following the all-in-one strategy (HAD group). Yellow arrows represented the perforation injuries, and Green arrows represented the HAD hydrogels. **(C)** Laparoscopic demonstration of forming rapid hemostasis and anti-adhesion barriers following the all-in-one strategy. Yellow arrows and circles represented the perforation injuries. Green arrows and circles represented the* in situ* HAD hydrogels.

We further validated the clinical translational potential of the all-in-one sutureless strategy for GP repair in porcine models (Figure 7A and Figure S18). In brief, we created artificial incisions (diameter = 5mm) on the stomachs of adult Bama minipigs by a biopsy punch to make acute gastric perforation models (Figure S19), and anesthesia was maintained with an inhalational anesthetic. The model group only received laparoscopy with no hydrogel treatment. For the all-in-one strategy treatment, the indocyanine green-labeled HAD precursor was precisely delivered to the gastric perforation area using the MID device through Channel 2 (C2). With the help of the MID device, it provided surgeons with a range of motion and haptic feedback in fluid-rich physiological environments (Video V6). Subsequently, HAD hydrogel formed robust tissue adhesion in body fluid-rich environments only within 10 s *via* the photocrosslinking of the MID device (Figure 7B), which offered the instant and sutureless repair of the micro and difficult-to-sew intraoperative perforations during the operation. In addition to the easy-to-apply sealing for micro intraoperative gastric perforations, we further demonstrated the ability of our all-in-one strategy for more complex clinical settings such as acute perforations with bleeding and gastric contents, which would tend to affect surgical visions for repair. Once the MID device was positioned at the gastric perforation sites, surgeons started UV transmitting *via* the MID device to form *in situ* gelation of HAD hydrogel barrier, triggering instant adhesion for rapid hemostasis and sealing of gastric perforation within 10 s (Figure 7C and Video V7).

**Figure 8:**
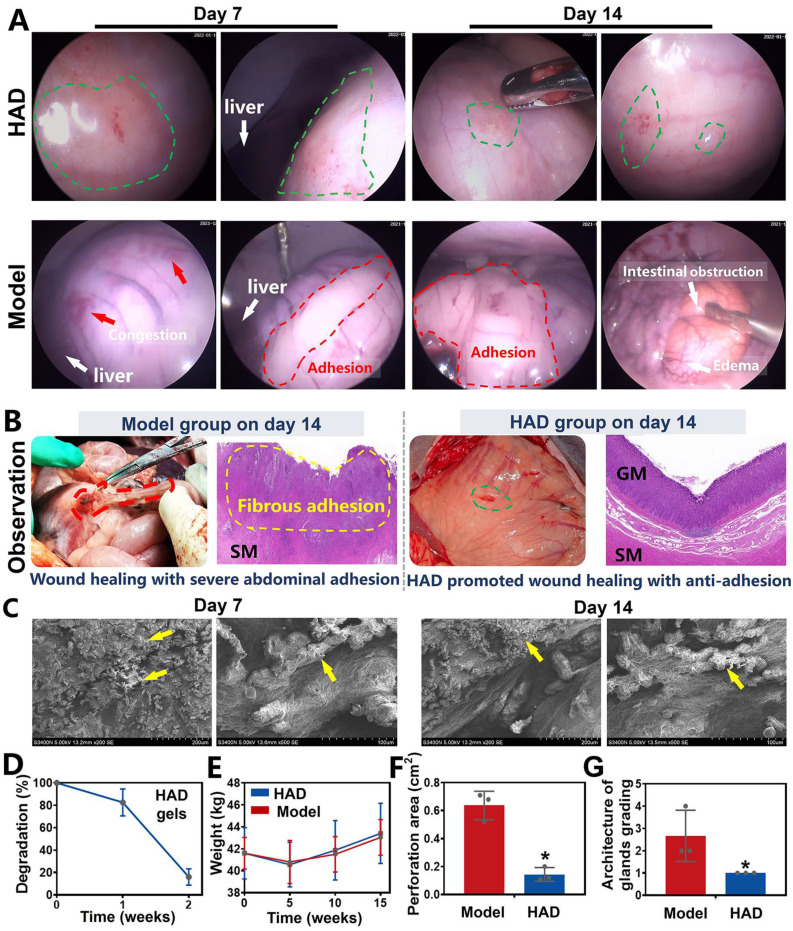
** The efficacy of the Janus HAD hydrogel in blocking gastric perforation and preventing postoperative adhesions in minipigs. (A)** Laparoscopic images of gastric perforations in the HAD and Model group after 7 and 14 days. Green circles represented the HAD hydrogel residues. Red circles represented the postsurgical abdominal adhesions. Red arrows represented the congestion on the stomach wall. **(B)** Macroscopic and H&E staining images of gastric mucosal tissue in the Model and HAD group on the 14th day after treatment. **(C)** HAD degradation products adhered to the perforation sites under SEM observation. **(D)** *In vivo* degradation rate of HAD hydrogels. **(E)** The average weight of minipigs in each group after surgery. **(F)** Area of gastric perforations in the model group and HAD hydrogel treatment group. **(G)** Histological evaluation of stomach sections based on the scoring of the architecture of glands (*P < 0.05 compared with the model group, n = 3 independent samples).

A 2-week laparoscopic surveillance was further performed to assess the efficacy of HAD hydrogels in blocking GP and preventing postoperative adhesions in minipigs, and the model group that only received incisional surgery was considered as the control. One week after laparoscopy, a clear contact interface between HAD hydrogels and the gastric wall was detected, providing continuous and stable protection of the injured tissues. Because the HAD hydrogel possessed numerous free catechol groups that strongly interacted with the natural nucleophiles in the proteins on the stomach surface. Further detection of deeper parts of the stomach showed that HAD hydrogels satisfactorily achieved effective hemostasis without fibrous adhesions between normal organs such as livers and abdomens (Figure 8A and Figure S20A). Detection two weeks after laparoscopy continuously revealed the complete healing of the perforations in the HAD group despite the obvious degradation of HAD hydrogels. Moreover, the HAD hydrogels degraded without any instance of postoperative adhesions between normal organs or even bowel instruction, as evidenced by laparoscopic photographs and video (Figure 8A, Video V8), which further indicated the protection of HAD prevented irreversible adhesions within 7-14 days after the surgery. In contrast, GP treated in the model group exhibited scattered congestion spots on the stomach surface and severe postoperative adhesions between abdomens and intestines 14 days after laparoscopy, as evidenced by laparoscopic photographs of the overall view of the pelvic cavity (Figure S20B). Severe adhesion tissues connecting the adjacent intestines and abdominal wall were observed in the model group on day 14 after surgery (Figure 8A). In contrast, Janus HAD hydrogels degraded 14 days after the operation, with the favorable repair of the perforation sites (Figure 8B). The HAD degradation products adherent to the perforation sites were confirmed under SEM, indicating stable wound protection and anti-postsurgical adhesions of Janus HAD hydrogels *in vivo* (Figure 8C-D). Additionally, the weight of the minipigs in the HAD group increased faster than those in the model group, indicating that HAD-treated pigs had better recovery of gastrointestinal function (Figure 8E). The macroscopic examination also revealed a significantly smaller perforation area in the hydrogel group than that in the model group (HAD group: 0.1433 ± 0.102 cm^2^ versus model group: 0.6367 ± 0.049 cm^2^; P < 0.05) (Figure 8F and Figure S21). H&E staining showed severe edema in the gastric mucosa of the model group compared to the HAD hydrogel group. Although epithelium regeneration was also observed in the model group, the structure of the glands was quite distorted. Compared with the model group, the degree of neutrophil and lymphocyte infiltration was significantly decreased in the HAD group. More regenerative epithelium with normal morphology and higher density of granulation tissue was also observed in the HAD group on day 14 (*P* < 0.05) (Figure 8G).

### Enhanced GP healing and anti-postoperative adhesion by the all-in-one repair strategy in minipigs

**Figure 9:**
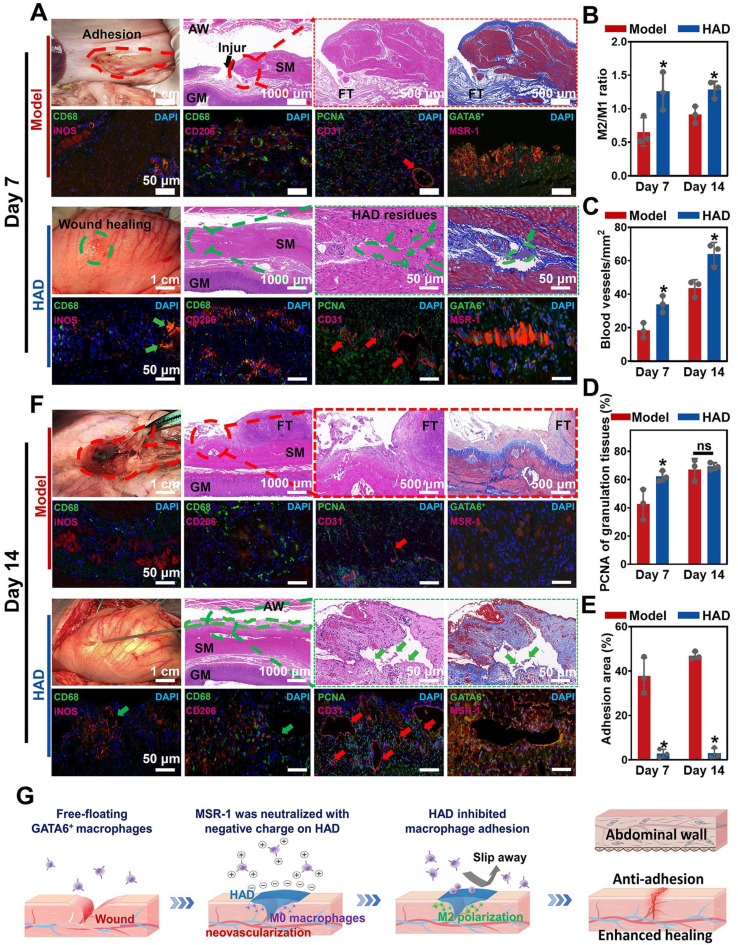
** HAD hydrogel promoted gastric mucosa healing for GP repair and inhibited uncontrolled recruitment of GATA6^+^ macrophage for anti-postoperative adhesion. (A)** Macroscopic photos and representative histological staining images in the model group and HAD group on day 7 after laparoscopy. Red circles represented adhesion tissues. Green circles and arrows represented the HAD hydrogel residues. Black arrows represented the perforation of injured tissues. Red arrows represented the new blood vessels. **(B)** The M2/M1 ratio in HAD and model group on day 7 and 14 after surgery. **(C)** Quantification of staining against the angiogenesis marker CD31 at HPF (original magnification: ×200) in HAD and model group on day 7 and 14 after surgery. **(D)** Quantification of staining against the PCNA around the wound margin at HPF (original magnification: ×200) in HAD and model group on day 7 and 14 after surgery. **(E)** Percentage of adhesion area in the model and HAD group on day 7 and 14 after surgery. (*P < 0.05 compared with the model group, n = 3 independent samples). **(F)** Photos and representative histological staining in the model group and HAD group on day 14 after laparoscopy. Red circles represented adhesion tissues. Green circles and arrows represented the HAD hydrogel residues. Red arrows represented the new blood vessels. **(G)** Schematic mechanism of HAD hydrogel for gastric mucosa healing and antiadhesion. Gastric perforation healing was observed with neovascularization and M2 macrophage polarization. Uncontrolled recruitment of GATA6^+^ cavity macrophages contributed to the adhesion formation, and the antiadhesion process of HAD was achieved by inhibiting uncontrolled aggregation of primordial GATA6^+^ macrophages by neutralizing MSR-1.

Minipigs in the model and HAD group were euthanized on the 7th and 14th day after laparoscopy. As shown in the macroscopic photographs on day 7, the model group revealed a scar formation and fibrosis covering the wound area (Figure 9A). From the H&E staining and Masson staining results, the structure of the gastric mucosa was quite distorted with deformed muscle in the model group, occurring with severe inflammation infiltration and fibrosis, and no obvious epithelium regeneration was observed. Compared with the model group, HAD-treated stomach tissues remained smooth with minimal fibrotic cyst formation. Residues of the Janus adhesive HAD hydrogels robustly adhered to GP sites, keeping interaction between hydrogels and tissues and helping bring the opposing mucosal edges at the perforation into the desired apposition (Figure 9A). Moreover, the HAD group showed fewer M1 macrophages (iNOS^+^ expression, an M1 marker) but a significantly higher number of M2 macrophages (CD206^+^ expression, an M2 marker) than that of the model group. The M2/M1 ratio in the HAD group was nearly 1.8-fold higher than that in the model group (P < 0.05), indicating that HAD hydrogels regulated the recruitment of marrow-derived macrophages by promoting M2 macrophage polarization (Figure 9A-B and Figure S22). Furthermore, re-epithelization and angiogenesis are the key factors contributing to gastric perforation repair 33, 34.

To assess neovascularization, CD31 (a marker for endothelial cells and angiogenesis) staining was assessed, and it showed that the number of CD31-positive capillaries (red arrow) in the granulation tissue in the HAD group was significantly higher than that in the model group (Figure 9A-C and Figure S23) (P < 0.05). Considering the impact of different treatments on reepithelization, HAD hydrogels induced significantly higher expression of PCNA (a proliferation marker of the G1-S phase) in granulation tissue than the model group on day 7 (P < 0.05) (Figure 9D and Figure S23), indicating that HAD hydrogels significantly accelerated the reepithelization of tissues around the gastric perforation. The perforation in the model group was repaired by fibrous tissues, predominantly composed of collagenous networks when compared with the HAD group. In addition, superaggregation of GATA6^+^ macrophages was observed around the perforation area in the model group (Figure 9A and Figure S24), whereas no adhesion or obvious aggravation of GATA6^+^ macrophages was observed at the HAD-treated perforation site due to the HAD polyanion formulation acting to neutralize scavenger receptors (Figure 9A and Figure S24). Correspondingly, the adhesion area of each group was evaluated by macroscopic examination, revealing a significantly smaller adhesion area in the HAD group than in the model group after 7 days (P < 0.05) (Figure 9E). Furthermore, on day 14, the model group exhibited undesired perforation healing with the progression of fibrotic adhesion. Although a clear recovery of the gastric mucosa was observed in the model group, the smooth muscle layer was still interrupted and accompanied by clear gaps in the injury sites that were larger than those seen in the HAD group (Figure 9F). In contrast, the degraded HAD hydrogel fragments bridged the regenerated gastric mucosa and the regenerative epithelium (Figure 9F). Moreover, the M2/M1 ratio in the HAD group was approximately 1.4-fold higher than that in the model group (P < 0.05) (Figure 9B and Figure S25), indicating that HAD hydrogels promoted the regeneration of gastric perforations with a continuous M2 polarization during the whole degradation process. In addition to the recruitment of M2 macrophages, the formation of new blood vessels for tissue regeneration in the HAD group was significantly higher than that in the model group on day 14 (P < 0.05) (Figure 8C and Figure S26). The number of PCNA-positive cells expressed around perforation margins in the HAD and model group both increased compared with that of day 7, there was no significant difference between the model and HAD group (P > 0.05) (Figure 9D and Figure S26). The degree of aggregation of GATA6^+^ cavity macrophages both in the model group and HAD group on day 14 was in agreement with the results of day 7. Subsequently, the adhesion area of the model group was enlarged, whereas no obvious peritoneum adhesion was observed in the HAD group after 14 days due to the blockage of MSR-1 proteins on GATA6^+^ macrophages (P < 0.05) (Figure 9E and Figure S27). In conclusion, the interaction between HAD hydrogels and perforation tissues indicated that HAD promoted wound healing through M2 macrophage polarization and the promotion of neovascularization. As for anti-postoperative adhesion, HAD hydrogels not only acted as a stable physical barrier but also as a polyanion trap to inhibit aggregation of GATA6^+^ Cavity macrophages. Taking advantage of the properties of HAD hydrogels and the MID device, the all-in-one strategy exhibited high efficacy in wound healing and anti-postoperative adhesions (Figure 9G).

## Discussion

In this work, we emphasized an all-in-one minimally invasive strategy for sutureless repair of acute gastric perforations, which consisted of injectable asymmetrically adhesive HAD hydrogel and the companion minimally invasive deliverable device, integrating bioadhesives and engineering delivery approaches into clinical practice. In our previous anti-adhesion research, we autonomously synthesized a dual-sided asymmetric adhesive HAD polyanionic hydrogel, which effectively prevents intra-abdominal adhesions. However, in the context of a more intricate and larger GP model, apart from necessitating a hydrogel capable of adhering to the complex wet surface of gastric perforation, there is also a need for a compatible device that enables real-time and precise delivery by surgeons during the procedure. Therefore, the primary innovation of this study lies in proposing an all-in-one minimally invasive repair strategy composed of a rapidly sealable gastric perforation, an adhesion-preventing HAD gel, and a corresponding minimally invasive delivery (MID) device. Following the all-in-one strategy, target delivery of HAD hydrogels *via* the MID device could achieve rapid and robust tissue adhesion in body dynamic environments, preventing a range of intraoperative and postoperative complications such as abdominal adhesions, fibrotic encapsulations, and bleeding. The injectable HAD hydrogels with asymmetrically adhesive properties overcame the disadvantages of double-sided adhesion that limited the application of most commercially available sealants for minimally invasive surgeries 16, 20. Before photocrosslinking, HAD precursors enabled adherence to the tissue surfaces due to the high reactivity between the abundant catechol groups on the HAD polymer chain and the natural nucleophiles (e.g., amido bonds, thiols, and amines) that are widely distributed in the proteins at the tissue surface 35, 36. In contrast, after photocrosslinking, the outer surface of the HAD hydrogels showed antiadhesion properties due to the establishment of the AEMA network limiting the free movement of catechol groups, thus inhibiting the reaction between the free-floating catechol groups on the HAD surface and the amino groups on the tissue surface. Moreover, HAD precursors demonstrated good shear thinning behavior, enabling them to conform to the complex and irregular surface of target tissues and not diffuse after implantation in the moist wound environment 14, 16. In the rat and rabbit acute gastric perforation models, the injectable HAD hydrogels proved the potential to improve wound healing through the suppression of inflammation, promotion of M2 macrophage polarization, and neovascularization, which led to the all-layer healing of the damaged stomach with regularly arranged and complete gastric mucosa. Additionally, a favorable effect against postoperative adhesion formation was also observed in the HAD-treated tissues. Abdominal adhesions develop in 79-90% of the patients who undergo open abdominal or pelvic surgery 37.

It has been reported that collagen deposition usually occurs around 7 days after surgery, which is an important sign of the formation of peritoneal adhesions 38. It indicated that MSRs of GATA6^+^ macrophages containing cysteine-rich domains can indiscriminately bind hundreds of different polyanionic ligands, and using polyanionic ligands as scavenger receptor antagonists would be an effective therapeutic strategy to inhibit the aggregation of GATA6^+^ cavity macrophages 16, 37. Intriguingly, HAD polyanion formulations acted as scavenger receptor antagonists to effectively inhibit aggregations of GATA6^+^ cavity macrophages, thereby preventing collagen deposition and postoperative adhesion formation. Hence, we believe that our injectable Janus adhesive HAD hydrogels have the potential for minimally invasive repair with favorable wound healing and anti-postoperative adhesion.

Despite the feasibility of being integrated into minimally invasive surgeries, the *in situ* gelation of HAD hydrogels needed the necessary photocrosslinking after HAD precursors were applied. Steps of HAD delivery and gelation were separated which might prolong the operation time to seal acute perforations with risks of postoperative complications such as adhesion formation and bleeding. To achieve rapid adhesion in body fluid-rich environments and simplify surgical manipulation, we designed the companion MID device integrating the hydrogel delivery and *in situ* photocrosslinking, which was able to function within one procedure through a single trocar channel. Although many studies have proposed the idea of integrating bioadhesives into minimally invasive manipulation, such as endoscopic delivery and balloon catheter treatment 1, 4, 20, 39, these biomaterials face a variety of problems. For example, spraying powder or oxidant solution via endoscopy to induce *in situ* gelation, could cause uncontrollable rejection or stimulation due to the complexities of delivering bioadhesives through narrow spaces and achieving strong adhesion in fluid-rich physiological environments 25, 40, 41. Moreover, for stapler-based minimally invasive delivery, which required compression against the tissue surface, the risks of leakage were high when applying staples to the closure of perforated tissues that demand very tight sealing, such as a traumatized stomach 39. Compared with the studies above, the MID device in this study integrated the function of target delivery and real-time *in situ* gelation of the HAD hydrogels, providing a specific and feasible solution for the rapid and fluid-tight sealing of acute perforations in the abdominal surgical emergency. With the MID device, the injectable Janus HAD hydrogels could be precisely delivered at any angle to any location visible by surgeons, overcoming the obstacle of handling the micro and difficult-to-sew intraoperative perforations, as evidenced by the surgical demonstration on the fresh human corpse.

The therapeutic efficacy of our sutureless all-in-one strategy was validated in the minipig acute perforation models. Following the all-in-one strategy, laparoscopic hemostasis and blocking acute gastric perforations could be achieved rapidly, and the application of HAD hydrogel barriers could be controlled in real-time by adjusting the degree of HAD hydrogels by surgeons. Due to the all-in-one strategy, the whole procedure of minimal manipulation on minipig models was performed through one trocar channel, leading to a decreased surgical time and a lower risk of trocar-site hernias 31, 42, 43. A continuous and well-integration contact interface between the Janus adhesive HAD hydrogels and perforations tissues was detected in the presence of blood or the moist body fluid at two weeks post-surgery, which effectively prevents gastric contents from leaking into the abdominal cavity. After implantation* in vivo* for 14 days, the injectable asymmetrically adhesive hydrogels degraded and repaired the perforated stomach well with all-layer healing due to the M2 polarization, significant neovascularization, and re-epithelization. Moreover, the outward non-sticky side of HAD hydrogels continuously and efficiently prevented the postoperative adhesion by day 14 and exhibited superior resistance to aggregation of peritoneal GATA6^+^ macrophages, thus significantly preventing *in vivo* fibrous capsule formation. Nevertheless, HAD still presents certain limitations. Before *in situ* gelation, the flowability of the HAD precursor solution could result in material loss on the wound surface, potentially impacting the accuracy and precision of wound sealing. Hence, the development of injectable Janus hydrogels with shear-thinning properties could offer a beneficial solution to address this concern.

## Conclusion

In summary, we successfully designed an injectable photocurable hydrogel with the potential of sutureless GP rescue and simultaneous anti-postsurgical adhesions. The lap shear test confirmed the wet adhesive properties of HAD hydrogel precursors on moist GP sites. Additionally, the bursting test conducted on ex vivo rat and porcine GP models demonstrated the robust and stable wet-adhesiveness of HAD, resulting from the synergistic effect of interfacial interactions and bulk toughness. This enabled sutureless and fluid-tight sealing of gastric leakages during emergencies. Encouragingly, the adhesion strength of HAD to the stomach remained stable after long-term immersion in PBS. Additionally, the outward surface of HAD was nonadherent after photocrosslinking, which endowed HAD with asymmetric adhesiveness to prevent post-operative adhesions during the GP repair. We further developed the compatible MID for precise delivery of HAD in laparoscopy. Moreover, experimental verification on minipigs has demonstrated the feasibility and potential use of the all-in-one sutureless repair strategy for laparoscopic gastric repair and anti-postsurgical adhesions. Using this strategy, the pigs survived the surgery, and the degraded HAD performed better than clinical sutures and conventional double-sided adhesion sealants. We believe that the injectable Janus HAD hydrogels, integrated with the MID device, offer a promising sutureless repair strategy for GP rescue and prevention of adhesions after minimally invasive surgeries.

## Supplementary Material

Supplementary figures and tables, video legends.

Supplementary videos.
