# Supplementary material for: Photocurable injectable Janus hydrogel with minimally invasive delivery for all-in-one treatment of gastric perforations and postoperative adhesions
Source: Theranostics. 2023 Sep 25;13(15):5365–85. doi: 10.7150/thno.87639 (PMC10614681; doi:10.7150/thno.87639)
Supplement: Supplementary file 1 — Supplementary figures and tables, video legends. [file thnov13p5365s1.pdf]

Supplementary Material

Photocurable injectable Janus hydrogel with minimally invasive delivery for all-in-one treatment of gastric perforations and postoperative adhesions

Xiaoqi Wu[1,2,4#], Zihan Wang[1,2#], Jie Xu[3], Liu Yu[1], Maoyu Qin[1], Jianfeng Li[1], Sitian Liu[1], Weihan Zheng[1], Zeyu Li[1], Jun Ouyang[1], Yanbing Li[1], Guoxin Li[2], Ling Wang[3*], Wenhua Huang[1*], Yaobin Wu[1*]*

[1] *Guangdong Engineering Research Center for Translation of Medical 3D Printing Application, Guangdong Provincial Key Laboratory of Digital Medicine and Biomechanics, Department of Human Anatomy, School of Basic Medical Sciences, Southern Medical University, Guangzhou, 510515, China*

[2] *Department of General Surgery, Nanfang Hospital, Southern Medical University, Guangzhou 510515, China*

[3] *Biomaterials Research Center, School of Biomedical Engineering, Southern Medical University, Guangzhou, 510515, China*

[4] *Department of Urology and Andrology, Ren Ji Hospital, School of Medicine, Shanghai Jiao Tong University, Shanghai 200001, China*

*** *Corresponding authors:*** *wuyaobin2018@smu.edu.cn (Y. Wu), hwh@smu.edu.cn (W. Huang), wangling0607@smu.edu.cn (L. Wang), liguoxin@smu.edu.cn (G. Li)*

[#] These authors contributed equally to this work.

Table S1. Comprehensive properties of recent hydrogel-based anti-adhesion barriers.

Hydrogels	Wet-adhesiveness (kPa)	Asymmetric adhesive	In *vivo* degradation (day)	Oxidation agent	Feasibility of laparoscopy	Gelation time (s)	X Anti-adhesion efficiency	Y= Maneuverability
(PAASP) hydrogel[1]	>5	√	>14	×	√	< 5	3	-1
Organ anastomosis glue[2]	>5	×	>14	-	√	< 5	1	2
TG-PTH hydrogel[3]	>5	×	>14	√	√	< 5	2	1
Janus patch[4]	> 20	√	7~14	×	×	-	-1	3
AOP127 /OHA[5]	4.6	×	> 14	×	√	> 10	1	-3
mIPN patch	> 20	×	7~14	×	×	-	1	0
ATgGel[6]	>5	×	7~14	-	-	< 3	-1	1
FJG powder[7]	>5	×	> 14	√	-	< 3	1	2
JPVA hydrogel[8]	> 15	√	> 14	×	-	< 3	3	2
mXG/HBC[5]	< 5	×	7~14	×	√	> 10	1	-1
(PEG)-based tissue glue[9]	> 15	×	5~7	-	-	> 10	-1	-1
ePTFE[5]	< 5	×	> 14	×	√	0	-3	-3
HAD + MID device (This study)	15	√	7~14	×	√	< 5	3	3

- If the wet-adhesiveness is less than 5kPa, the score of hydrogels was -1, otherwise it is 1;
- If the hydrogel is not asymmetrically adhesive, the score of hydrogels was -1, otherwise it is 1;
- If the *in vivo* degradation time is between 7~14 days (the peak of postoperative adhesions), the score of hydrogels was 1, otherwise it is 1;
- If the gelation of hydrogels needs oxidation agent, the score of hydrogels was -1, otherwise it is 1;
- If the hydrogel is injectable, the feasibility of laparoscopy scored 1, otherwise it is -1;
- If the time of gelation is over 30s, the score of hydrogels was -1, otherwise it is 1;
- Y: Maneuverability of laparoscopic surgery=the sum score of "oxidation agent", the feasibility of laparoscopy" and "time of gelation".
- X: Anti-adhesion efficiency = the sum score of "wet-adhesiveness", "asymmetrically adhesive" and "*in vivo* degradation time".

Table S2. The standard scoring system was used to evaluate the degree of adhesion.

Score	Peritoneal adhesion
0	No adhesion
1	One thin filmy adhesion
2	More than one thin adhesion
3	Thick adhesion with the focal point
4	Thick adhesion with plantar attachment or more than one thick adhesion with focal point
5	Very thick vascularized adhesion or more than one plantar adhesion

Table S3. Sequences of primers used in RT-qPCR

Primer	Sequence (5'-3')
**TNF-α (forward primer)**	ATGGGCTCCCTCTCATCAGT
**TNF-α (reverse primer)**	GCTTGGTGGTTTGCTACGAC
**TGF-β1 (forward primer)**	TCGACGTTTGGGACTGATCC
**TGF-β1 (reverse primer)**	AGTGGCTGAACCAAGGAGAC
**IL-6 (forward primer)**	AGAGACTTCCAGCCAGTTGC
**IL-6 (reverse primer)**	CTGGTCTGTTGTGGGTGGTA

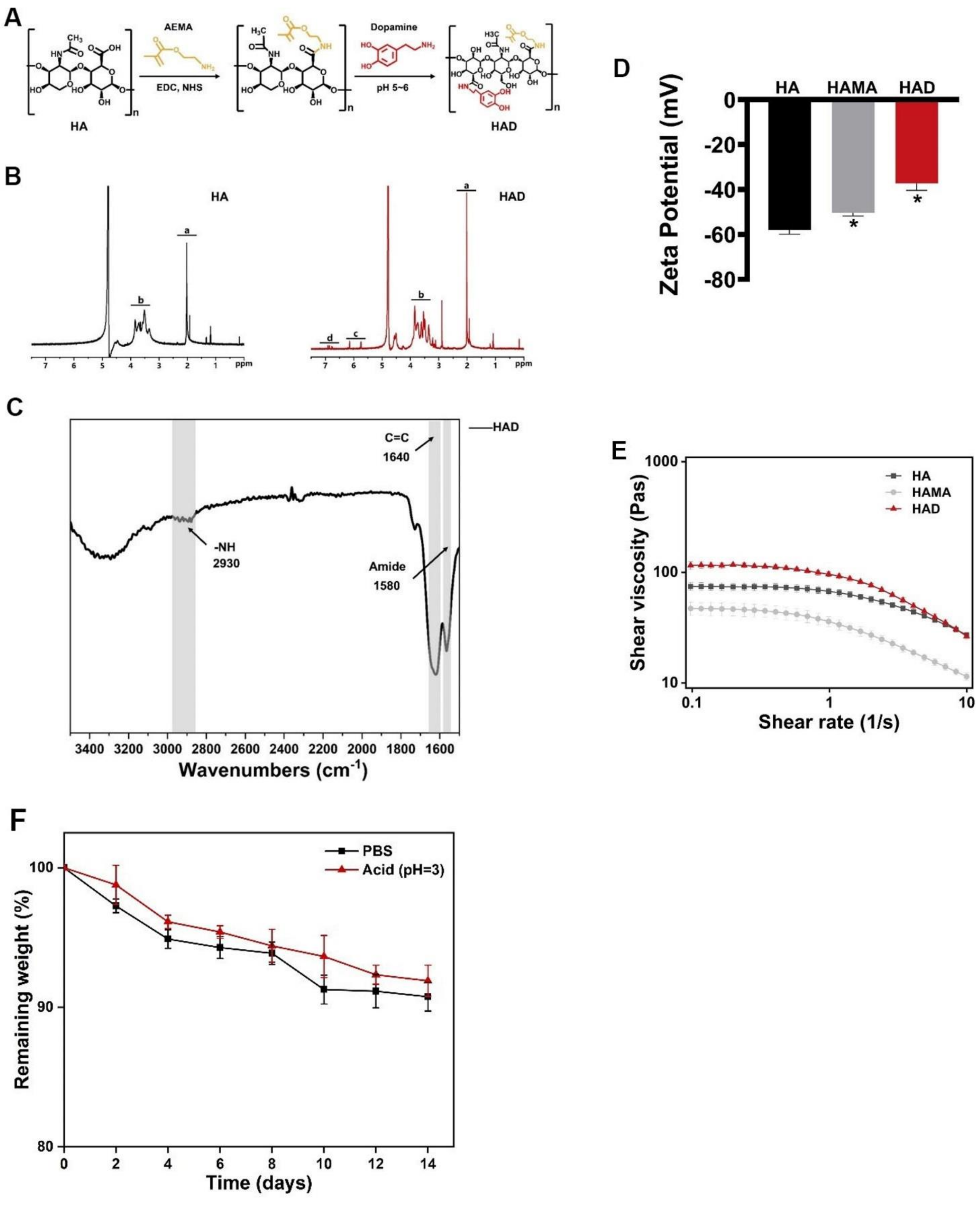

Figure S1. (A) Schematics on the synthesis of the HAD formulation. **(B)** ^{1}HNMR spectra of HAD formulations. **a** labeled the C(=O)CH3 in HA (δ =2.1 ppm); **b** labeled protons in the ring structures of HA (δ =4.0 ~3.0 ppm); **c** labeled the C=CH2 of 2-aminoethyl methacrylate hydrochloride (AEMA)

(δ=5.68 and 6.13 ppm); **d** labeled protons in the catechol ring of dopamine(DA) (δ=6.5~7.2 ppm). **(C)** For the FT-IR spectra of the HAD formulations, the appearance of absorption bands at 1580 cm^{-1} and 1640 cm^{-1} were assigned to the amide groups, vinyl groups, respectively. **(D)** Zeta potential of HAD precursor and HA solution at pH7.4. *$p<0.05$ compared with the HA group. **(E)** The shear viscosity test was carried out by increasing the shear rate from 0.1 to 10 s^{-1}. **(F)** Degradation behavior in vitro of HAD hydrogels.

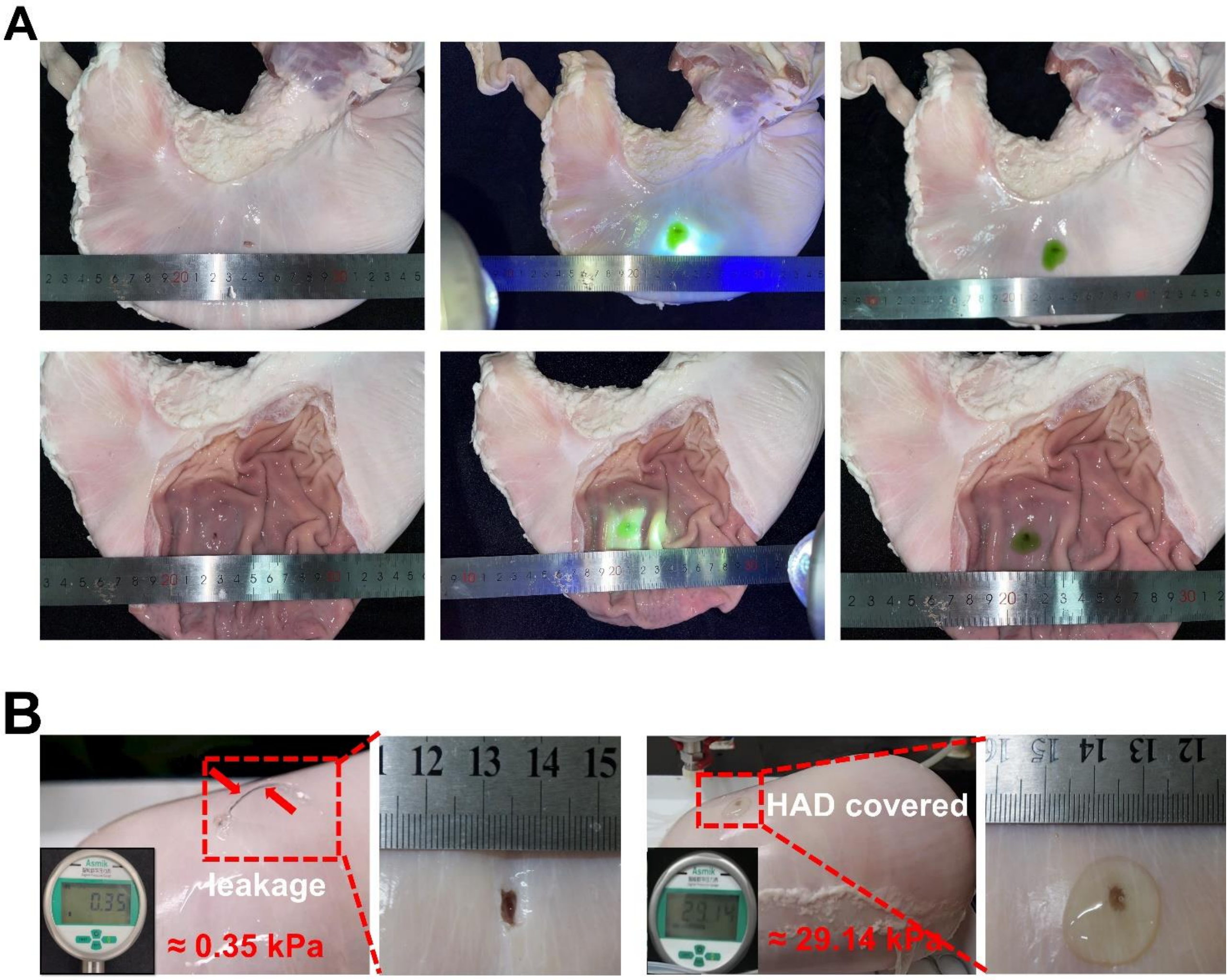

Figure S2. (A) HAD hydrogel was able to adhere to both outside and the inside wall of the porcine stomach after 5s of photocrosslinking. (**B)** HAD successfully formed tight-fluid sealing and withstood an exaggerated deformation.

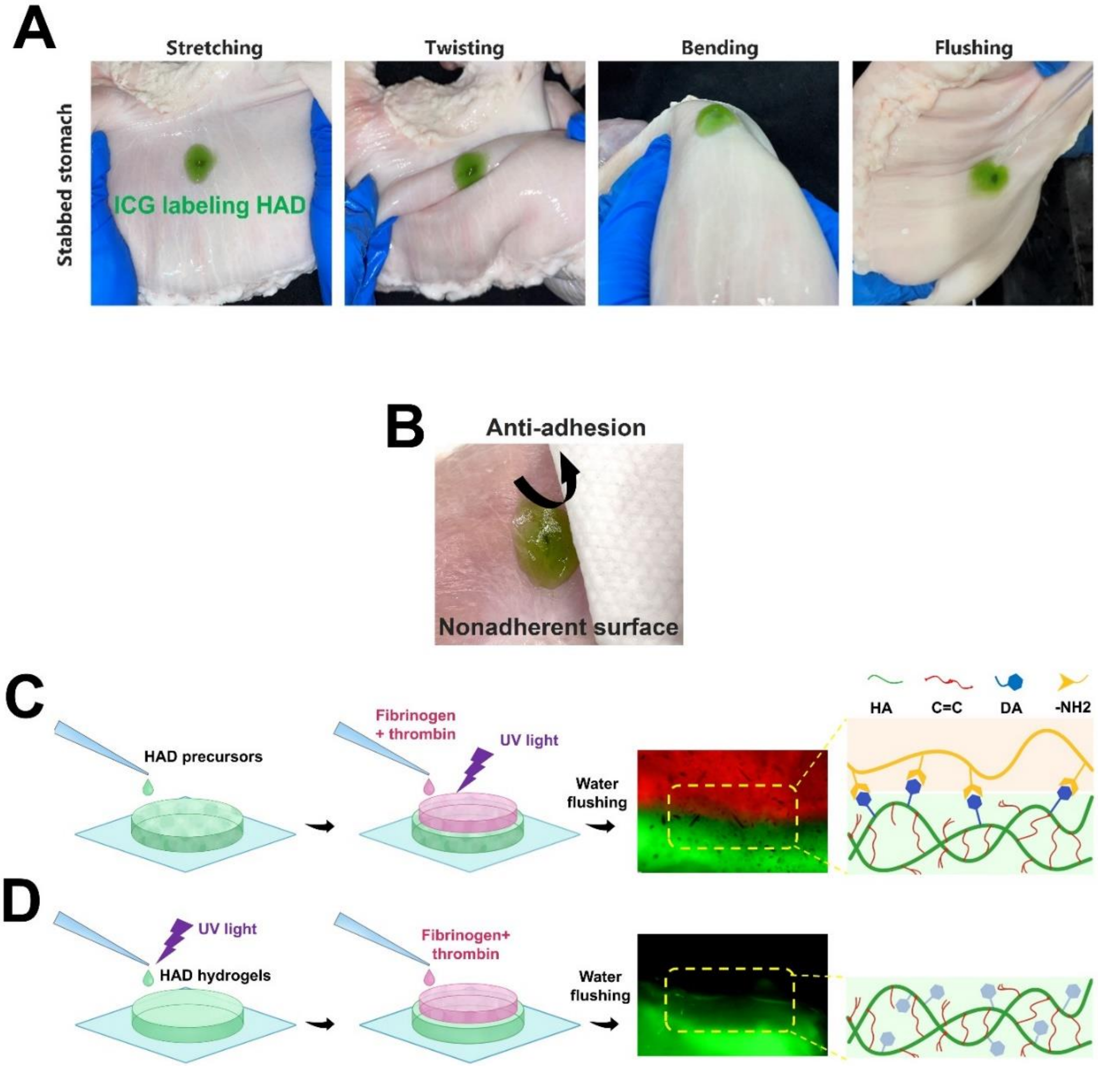

Figure S3. (A) HAD hydrogels robustly adhered to the ex vivo porcine perforation under the torsion for different angles. **(B)** The outside face of HAD hydrogel was nonadherent to the paper napkin. **(C)** Fibrin adsorption on the surface of the HAD hydrogels following the "sol-adhesive" process. The fibrin labeled with red (Alexa Fluor 594-conjugated), and the HAD formulation labeled with green (mixed with FITC dye). **(D)** Fibrin failing to adhere to the surface of crosslinked HAD following the "gel-nonadhesive" process.

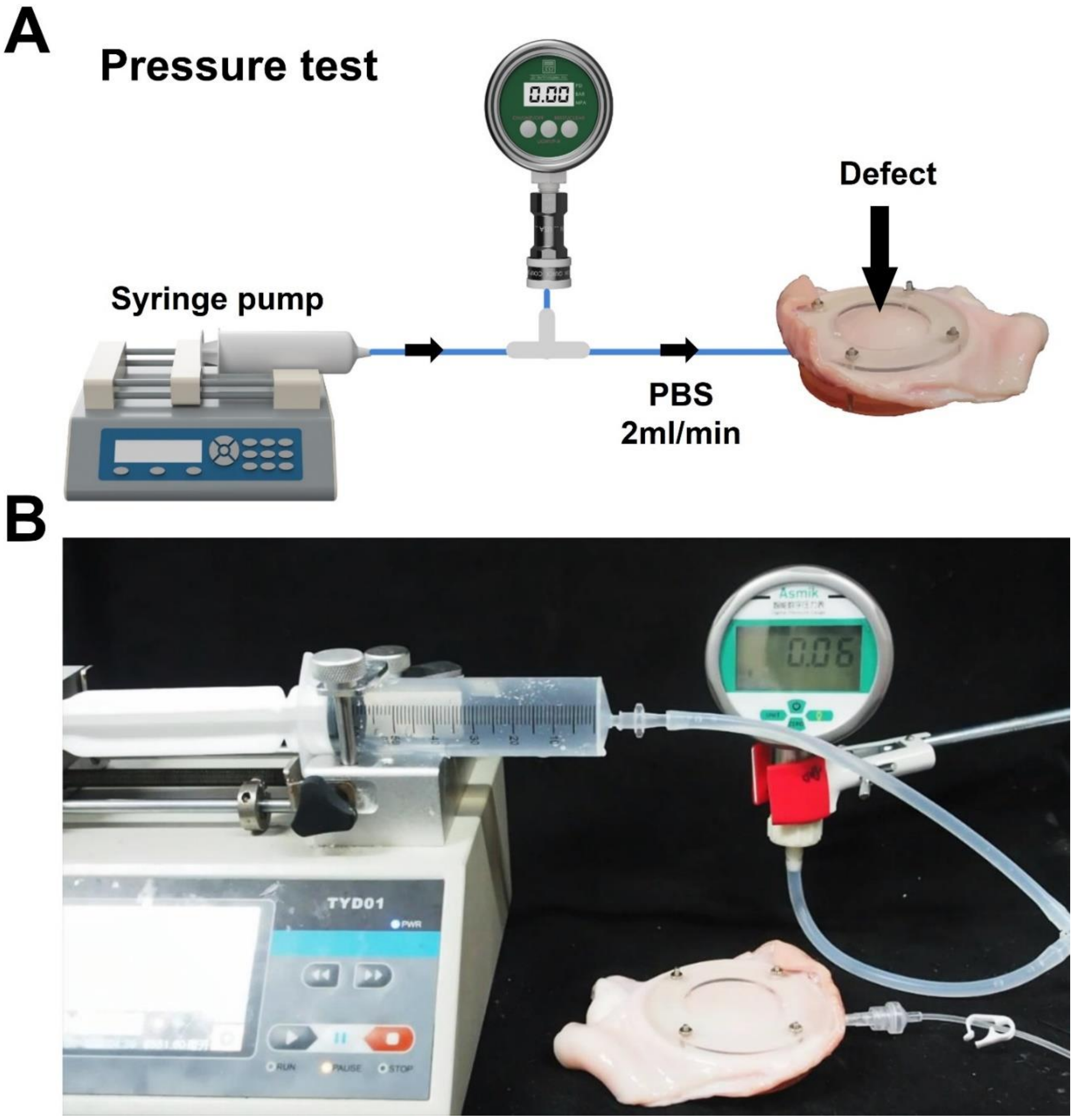

Figure S4. (A) Schematic diagram of the bursting press test. (**B)** Practical diagram of the bursting press. test.

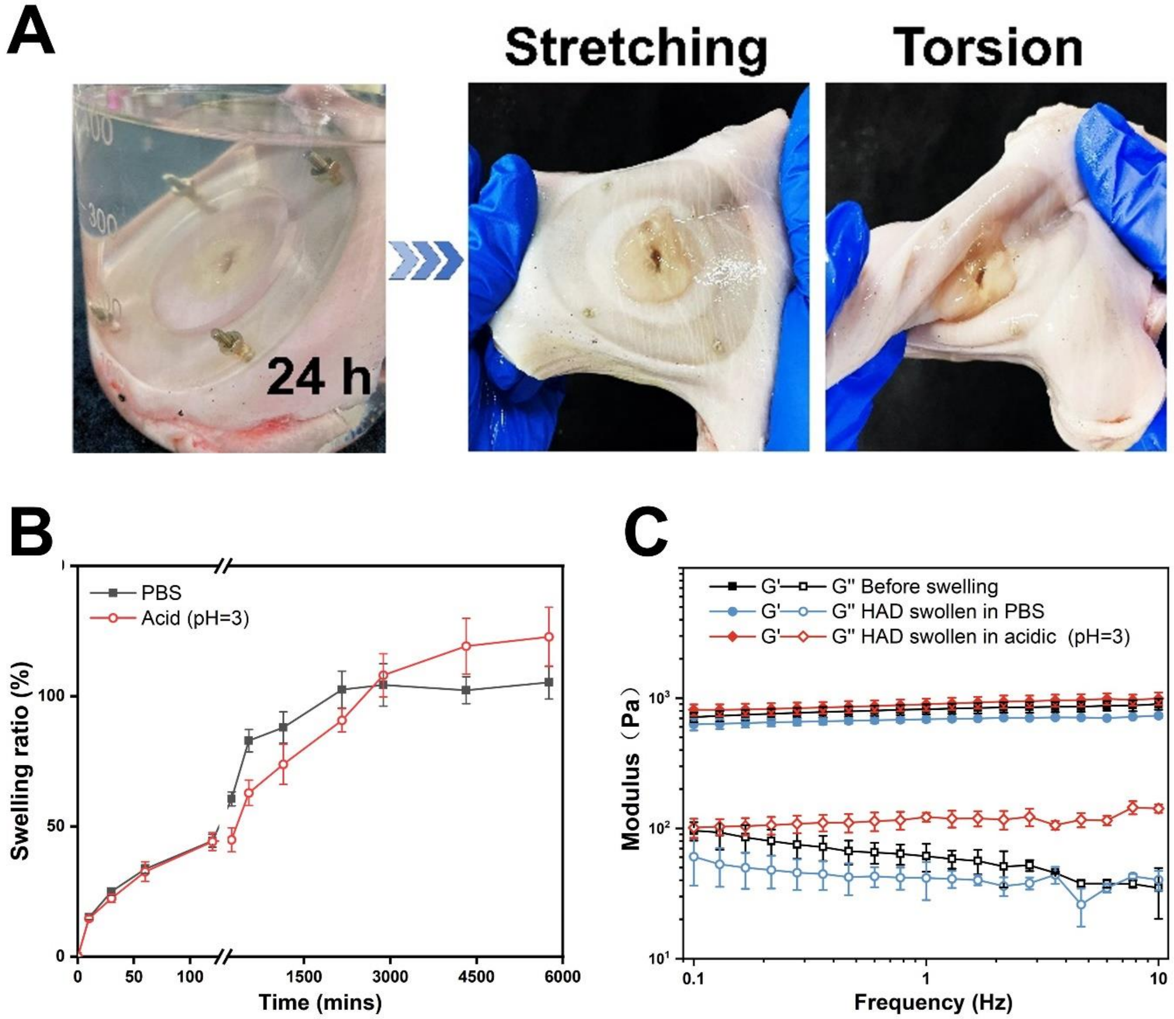

Figure S5. (A) HAD hydrogels still robustly adhered to the ex vivo porcine perforation when imposed with stretching and torsion after immersion in PBS for 24h. **(B)** *Ex vivo* **s**welling ratio properties of HAD hydrogels in PBS and Acid (pH=3). **(C)** Rheological properties of HAD hydrogels with the frequency-sweep (0.1–10 Hz, strain of 1%) test.

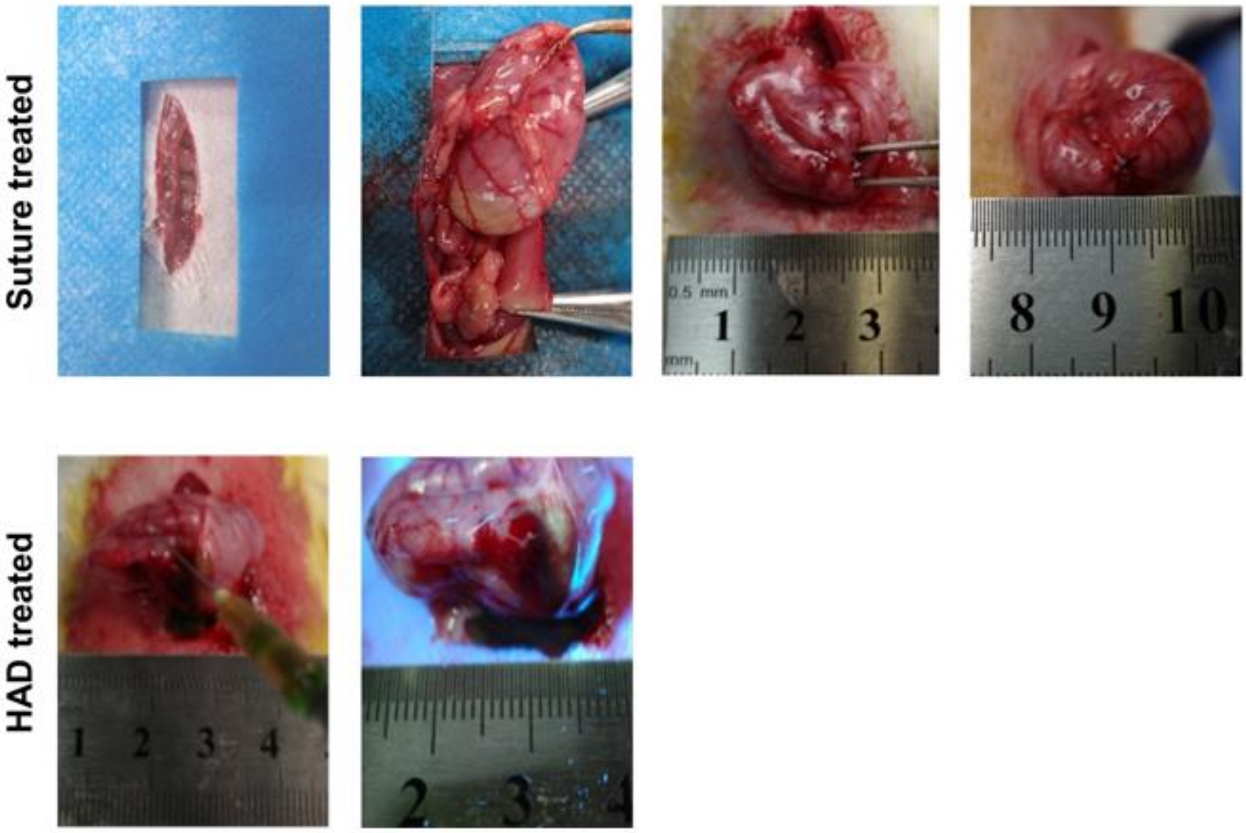

Figure S6. Procedures of establishing an artificial defect on the rat stomach, which was treated by surgical sutures and HAD hydrogels respectively.

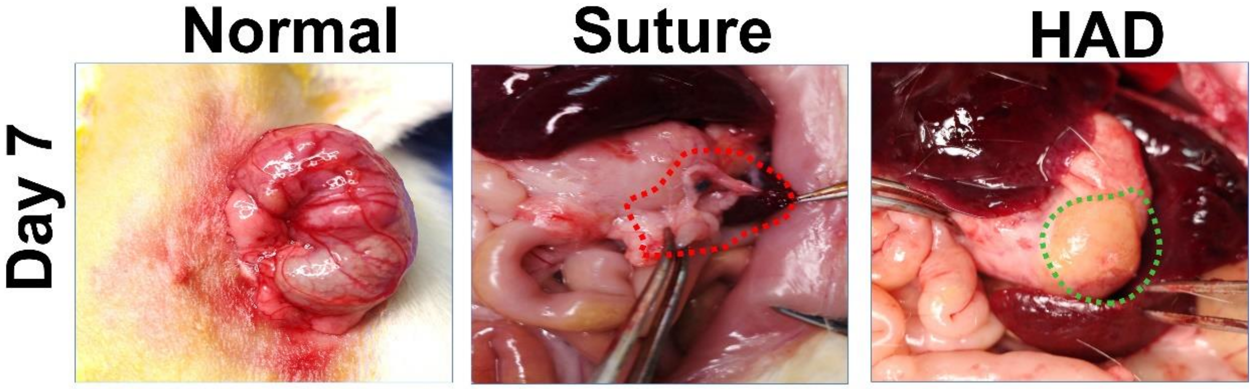

Figure S7. Macroscopic photo of rat gastric wounds in each group 7 days after surgery. Adhesion tissues were circled in red lines and HAD hydrogel residues were circled in green lines.

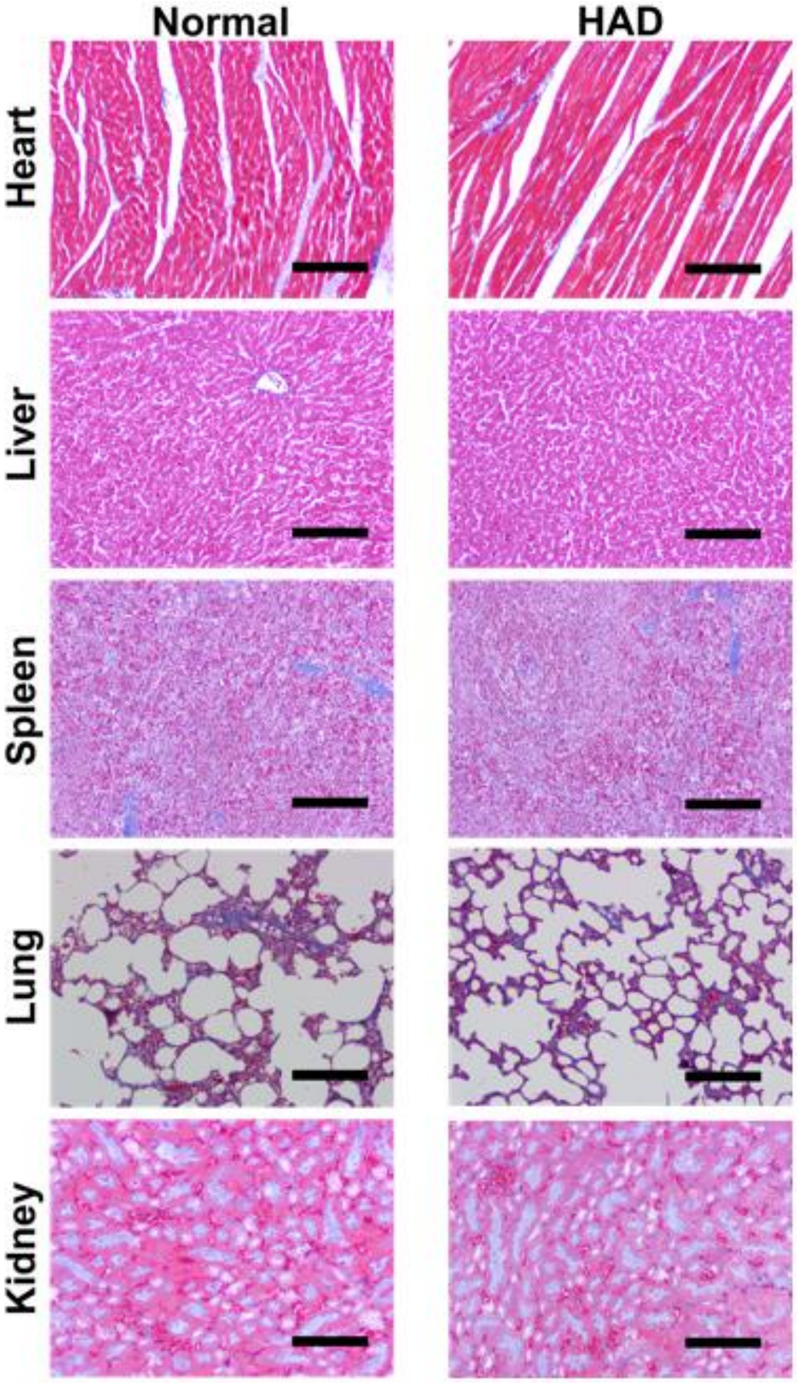

Figure S8. Representative H&E staining images of tissue sections (heart, liver, spleen, lung, and kidney) demonstrated the biocompatibility in vivo of HAD hydrogels. The complete degradation of HAD hydrogels was observed without obvious symptoms of inflammation, such as exudation, condensation, or infiltrate during the 14 day-period of gross and pathological observation. Scale bars = 400 μm.

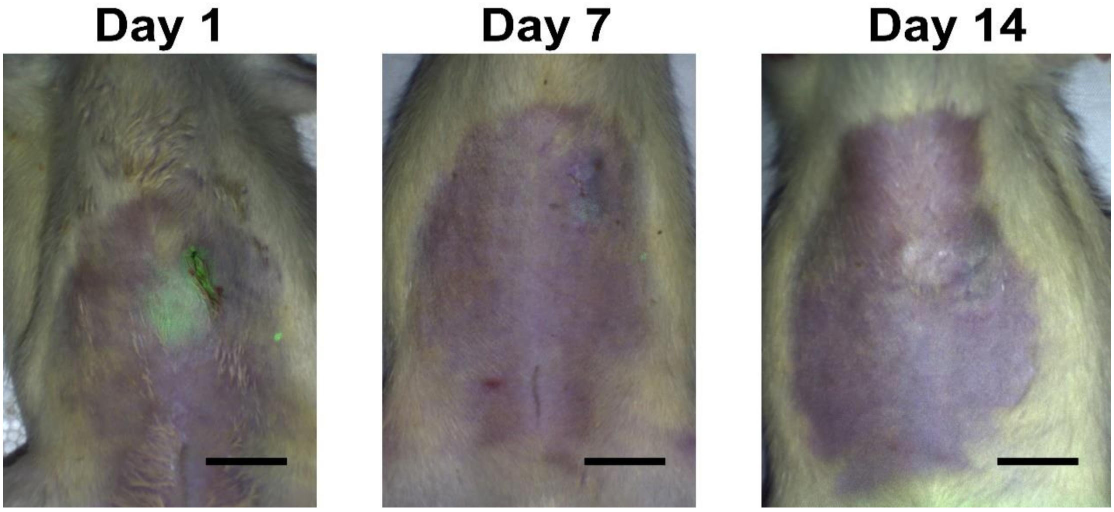

Figure S9. Fluorescence images of ICG stained HAD hydrogels on 1, 7, and 14 days after the implantation on the gastric operation. Scale bars = 1.5cm.

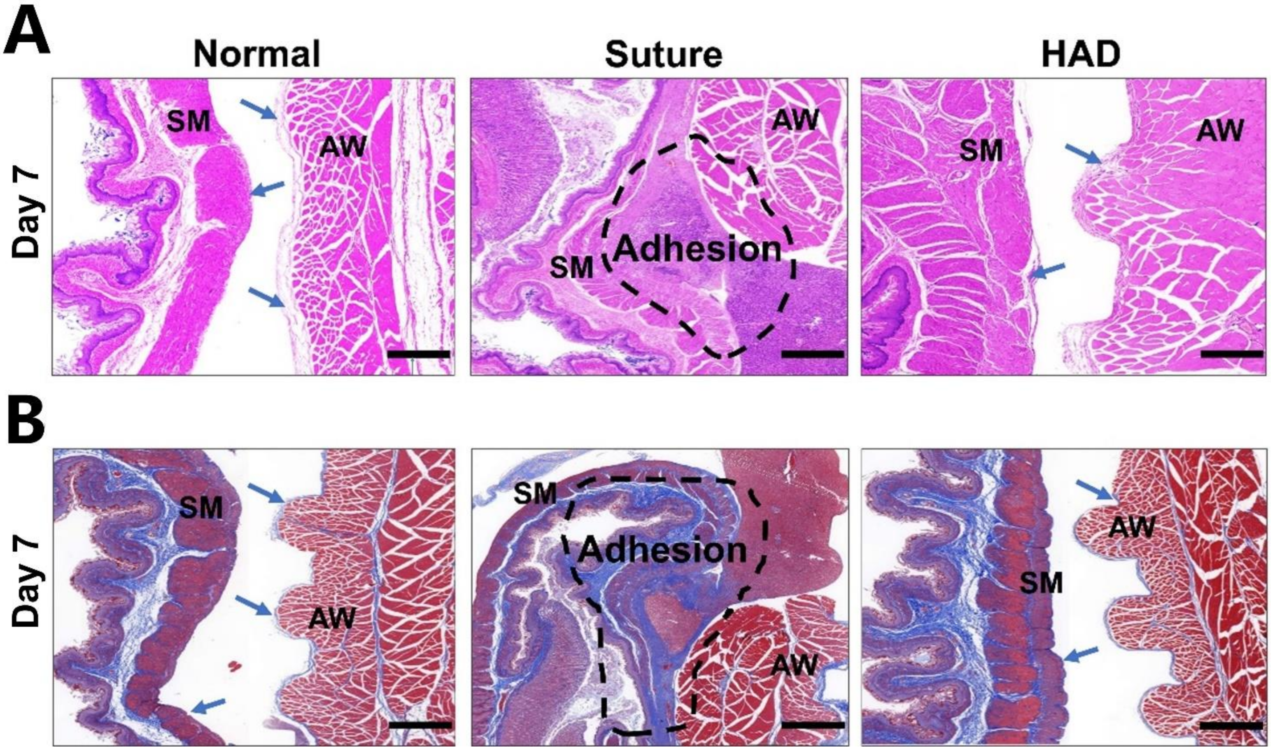

Figure S10. (A) Representative H&E staining of tissue sections in different groups after abdominal surgery for 7 days. (**B)** Masson's trichrome staining images of tissue sections in different groups after abdominal surgery for 7 days. AW, abdominal wall; SM, smooth muscles; The blue arrows pointed to mesothelial cells. Adhesion tissues are marked with black circles. Scale bar = 200 μm.

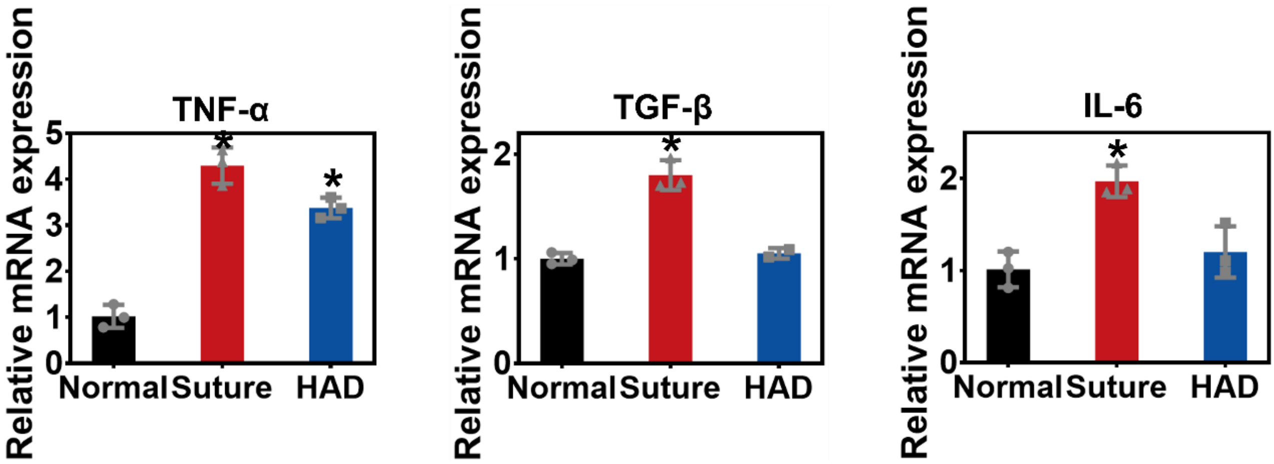

Figure S11. Related mRNA expressions of TNF-α, TGF-β1, and IL-6 in the Normal, Suture and HAD groups on day 7 after the first surgery. **P*< 0.05 compared with the Normal group.

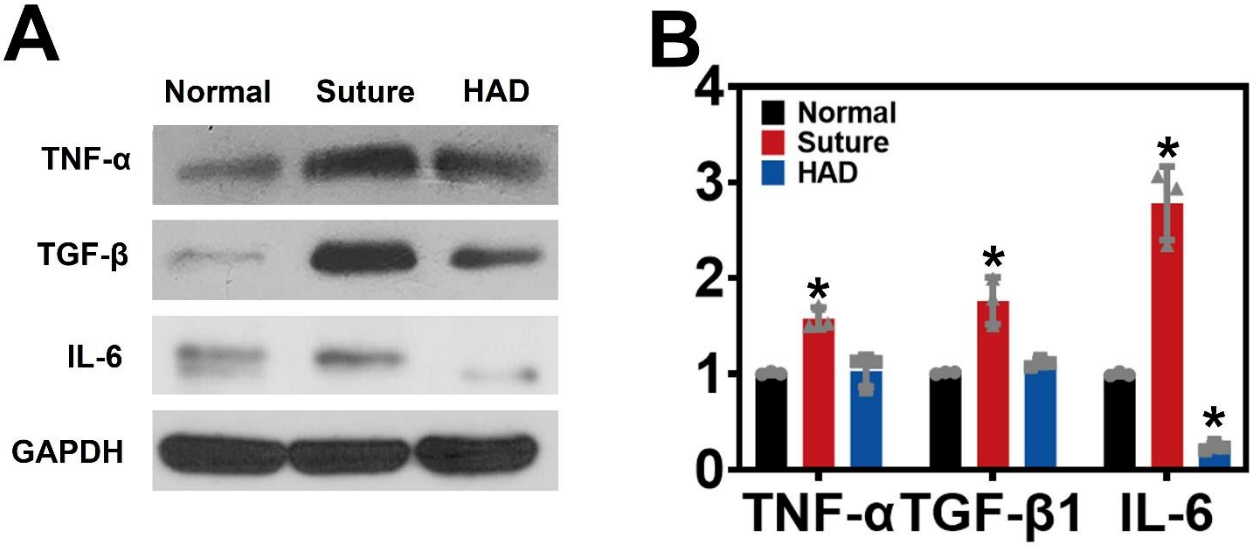

Figure S12. Expression levels of TNF-α, TGF-β1, and IL-6 were validated by Western blotting. (**A)** The WB assay of Normal, Suture and HAD group on day 7. (**B)** All WB bands were normalized to the GAPDH bands. **P*< 0.05 and ***P*< 0.01compared with the Normal group.

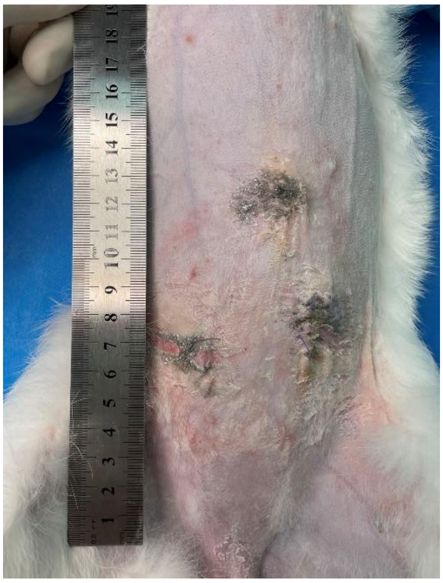

Figure S13. Gross observation of rabbits treated by the laparoscopy on day 14. Obvious trocar induced injuries was observed on the abdominal wall.

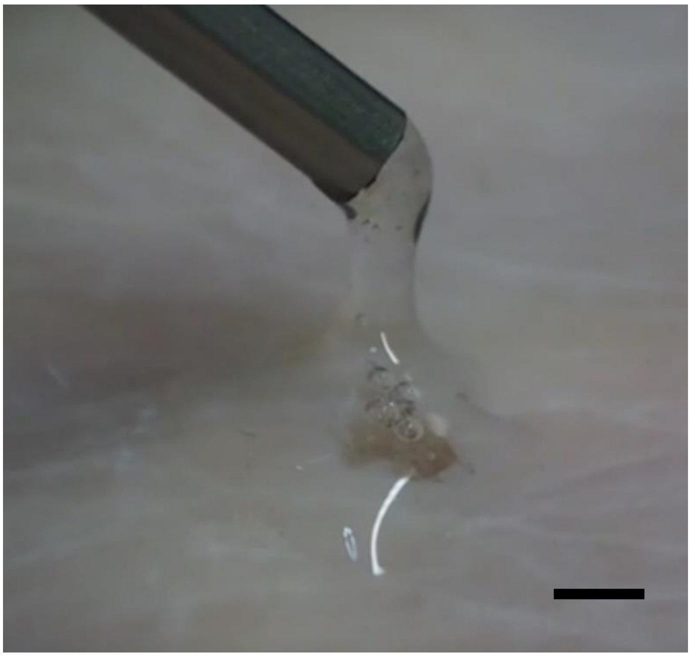

Figure S14. The practical operations of sealing gastric defects via the MID (minimally invasive integrated device. If the needle was not retractable, the contamination of the optical fibers could not be avoided during the process of injecting hydrogels in the target sites, which was detrimental to the UV transformation. Scale bar: 5mm.

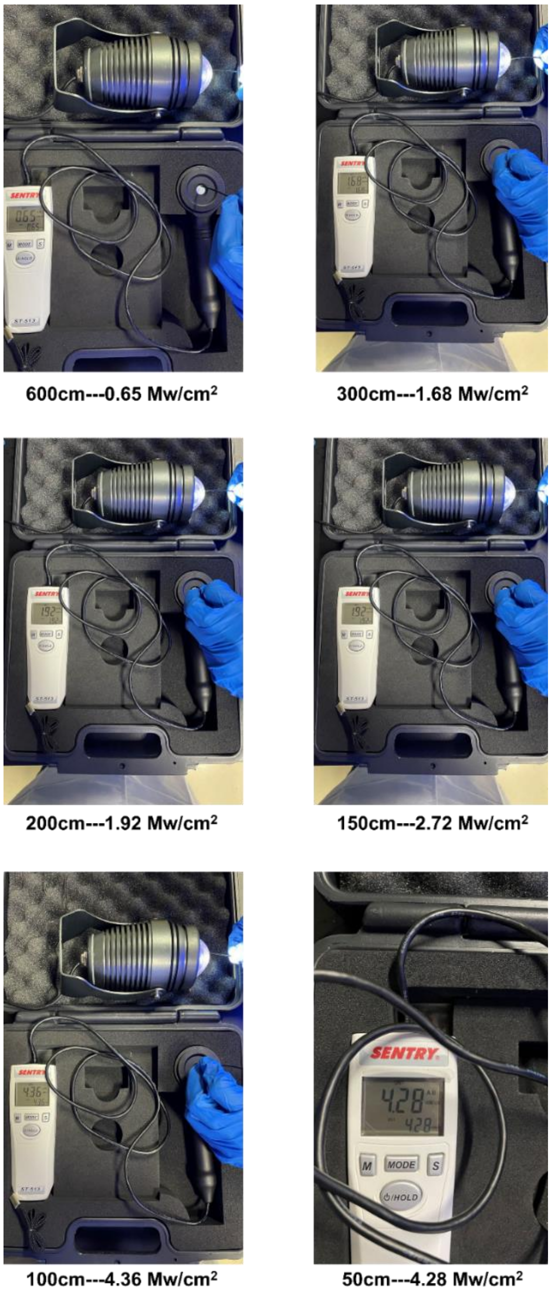

Figure S15. The relationship between fiber length and UV intensity. Specifically, the UV light intensities decreased exponentially as the diameter of the fiber was increased. The similar result was observed in the relationship between the fiber length and UV light intensities showing that a shorter length of the fiber resulted in stronger UV light intensities. When the length of fiber is decreased to 50cm, the UV light intensity was kept at about 4 mW/cm^2, which was the minimum threshold UV power intensity to form the HAD hydrogels.

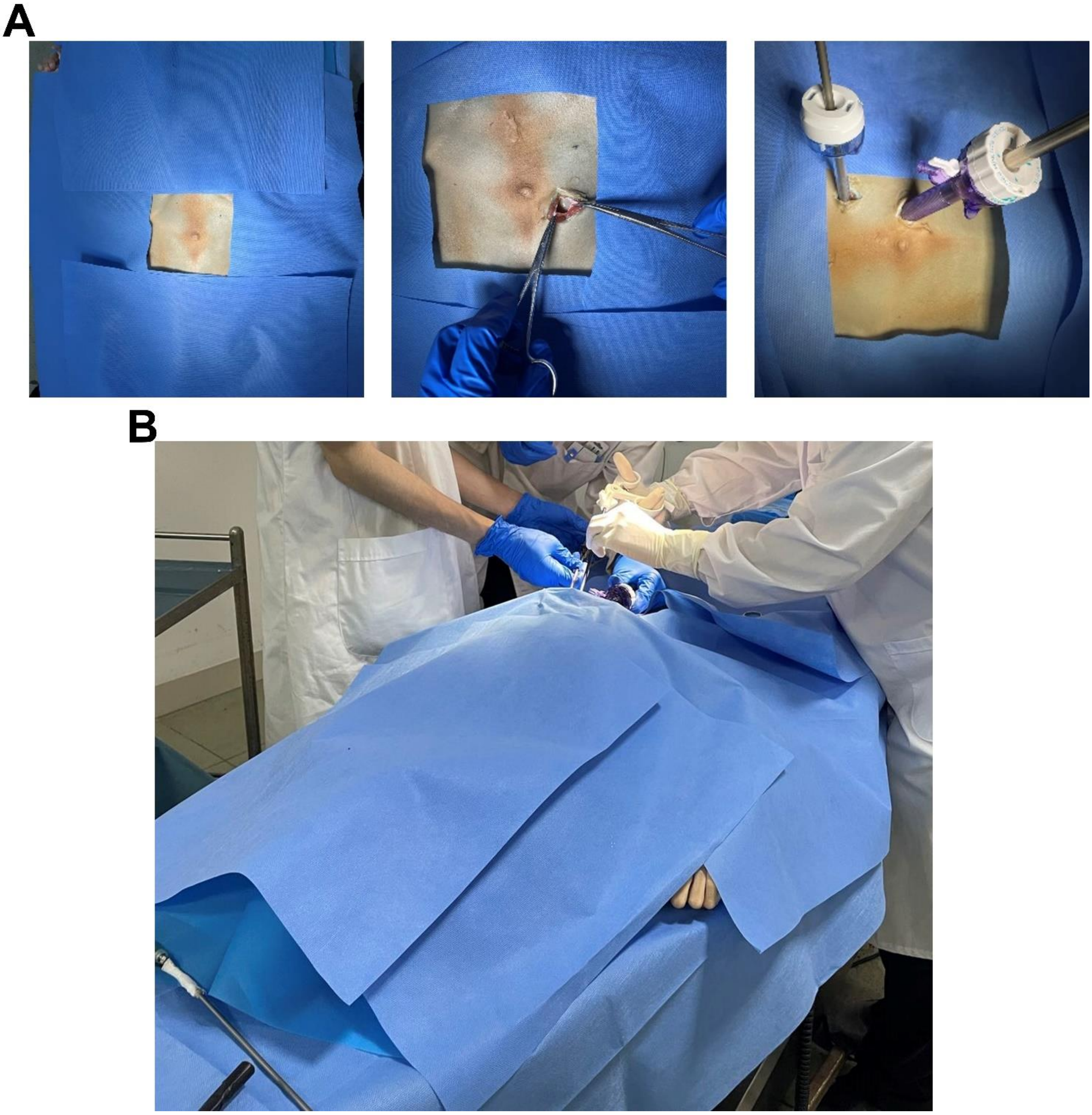

Figure S16. The preoperative preparations of applying the minimally invasive integrated device (MID) in the fresh human corpse. The fresh human corpse was approved by Southern Medical University Ethics Committee and Medical Ethics committee of Nanfang Hospital (Ethical authorization number is NFEC-2022-381.)

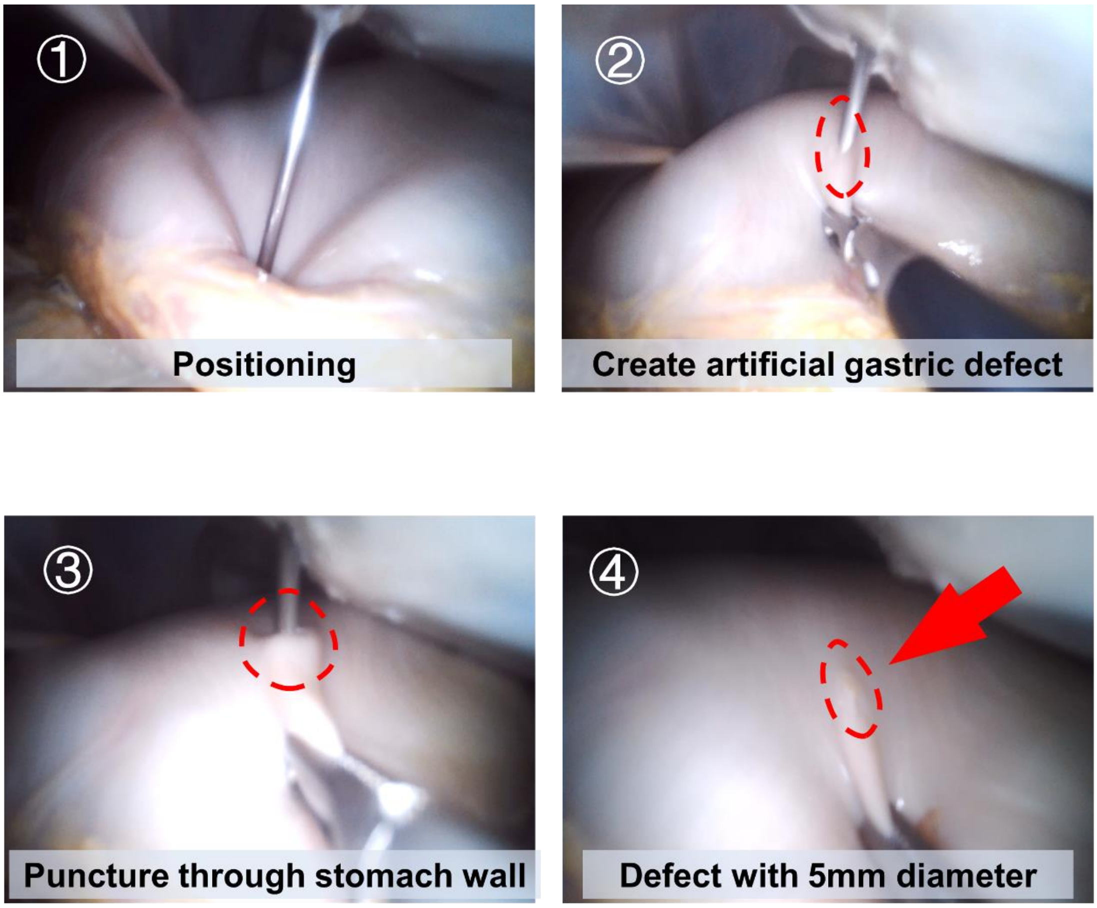

Figure S17. The preoperative preparations of applying the HAD hydrogels via the MID in the fresh human corpse.

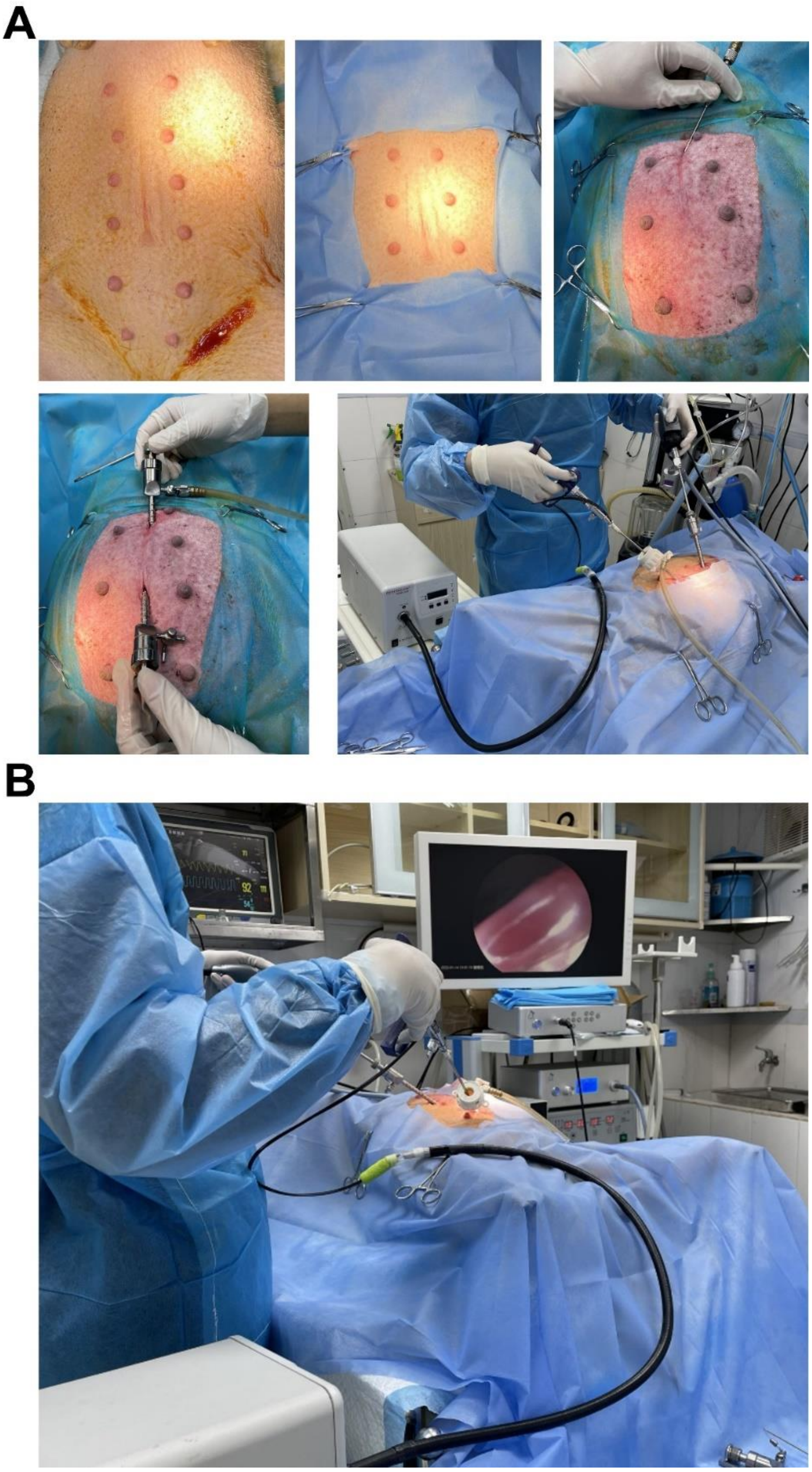

Figure S18. (A) The preparations for the minipig acute gastric models. Anesthesia was maintained with an inhalational anesthetic, and the a pneumoperitoneum was established on the abdomen. (**B**) The general view of the laparoscopy on minipigs using the MID.

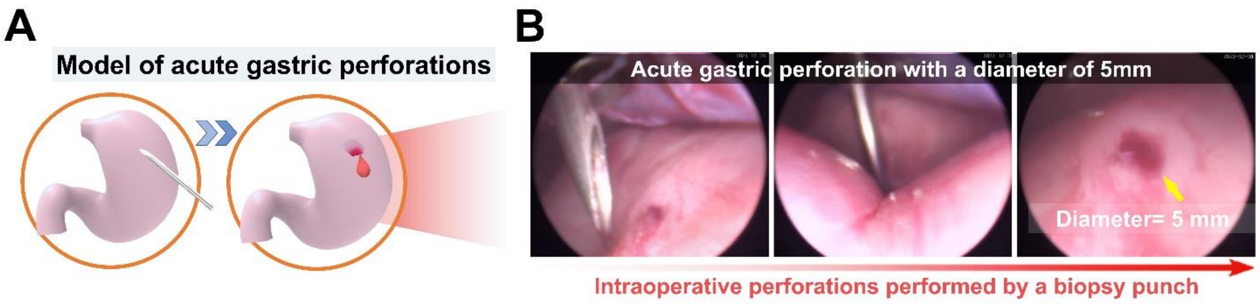

Figure S19. Schematic illustration (A) and laparoscopic imaging (B) of acute perforation models with 5 mm defects (model group), and the yellow arrow represented the perforation injuries.

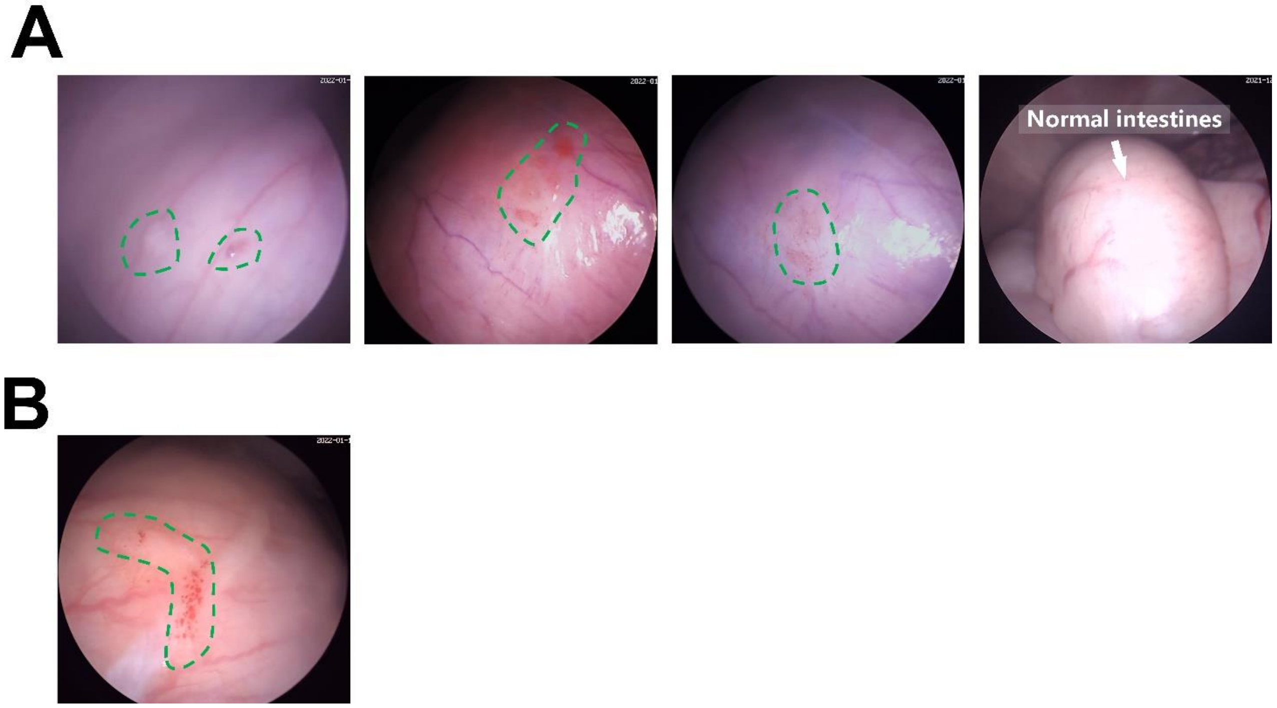

Figure S20. Laparoscopic images of gastric perforations in the HAD (**A**) and Model group (**B**) on day 14.

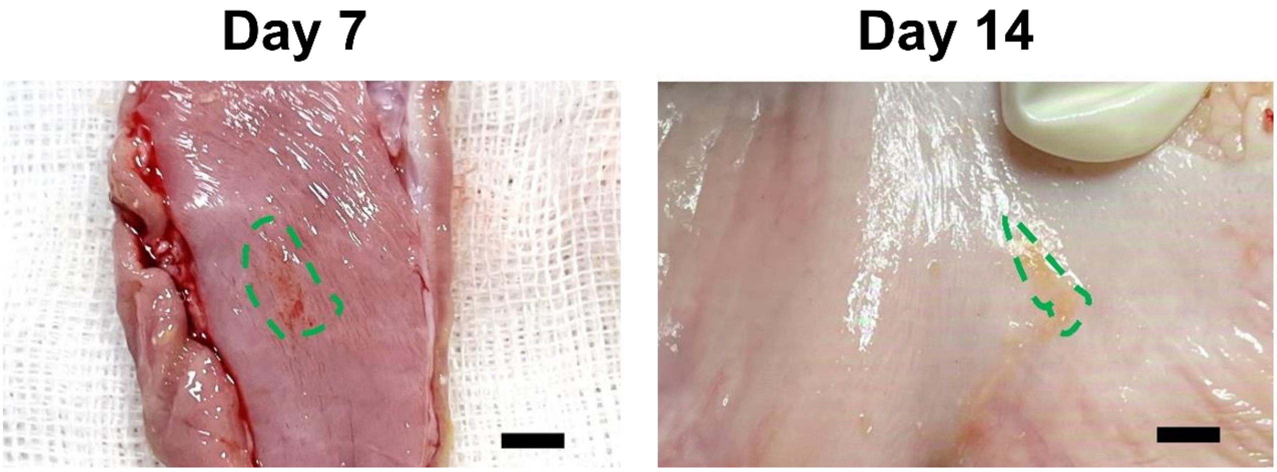

Figure S21. The porcine gastric tissues were harvested for further histopathological examination on day 7 and 14 after the laparoscopy. Scale bar = 5mm.

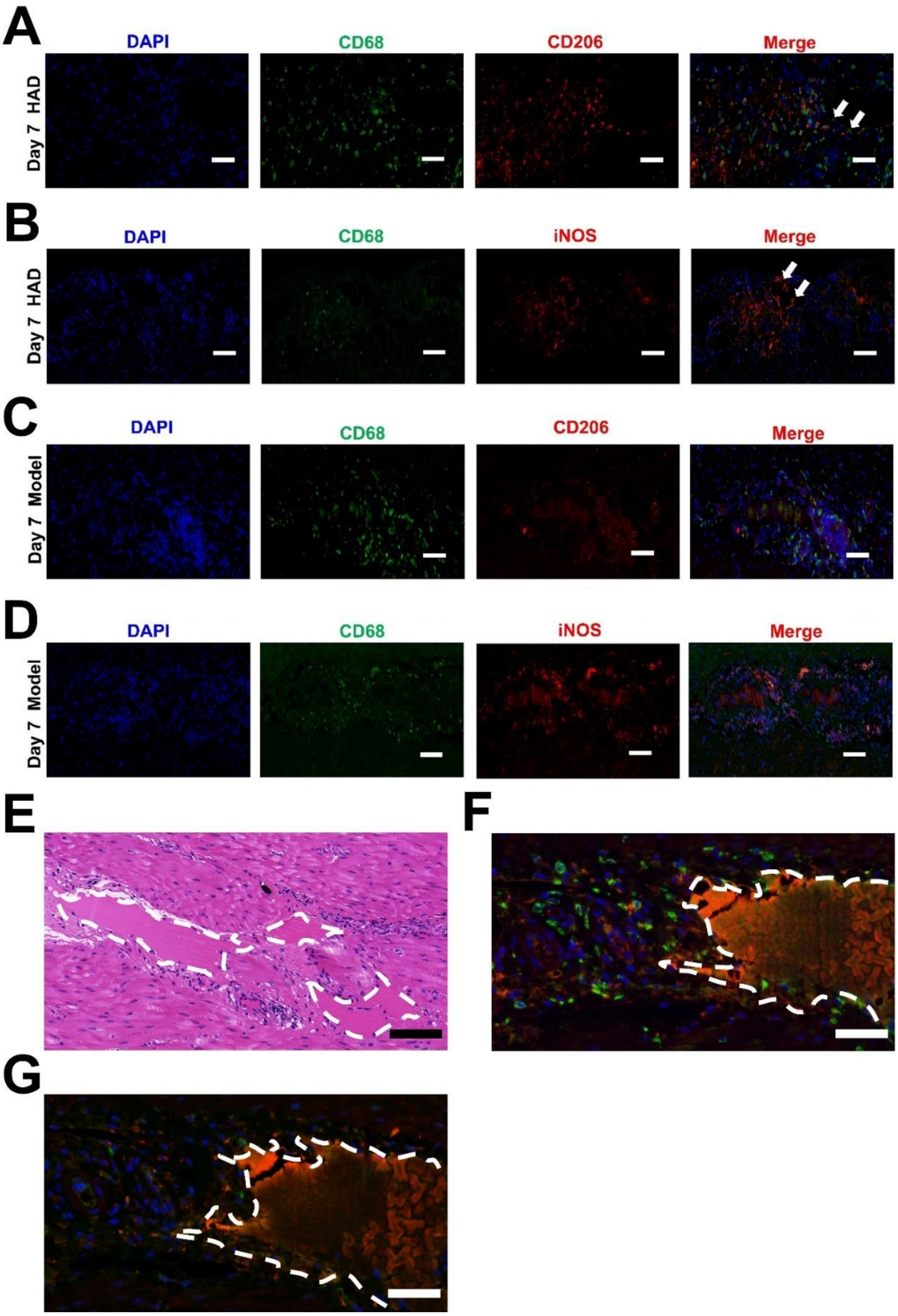

Figure S22. (A) Immunofluorescence staining of CD206 (red) and CD68 (green) in HAD group on day 7 after surgery. (B**)** Immunofluorescence staining of iNOS (red) and CD68 (green) in HAD group on day 7 after surgery. (**C)** Immunofluorescence staining of CD206 (red) and CD68 (green) in Model group

on day 7 after surgery. (**D)** Immunofluorescence staining of iNOS (red) and CD68 (green) in Model group on day 7 after surgery. The white arrows represented the residues of HAD hydrogels. Scale bars = 50 μm. **(E)** In vivo HAD degradation fragments after 7 days did not cause inflammatory cell infiltration in the muscular layer of the gastric perforation (400x magnification of the gastric perforation site). **(F)** Following 7 days of in vivo degradation, HAD induced M2 macrophage polarization (CD68 labeled in green, CD206 labeled in red, 400x magnification of the gastric perforation site). **(g)** Degree of M1 macrophage polarization promoted by HAD after 7 days of in vivo degradation. (CD68 labeled in green, iNOS labeled in red; 400x magnification of the gastric perforation site). The white arrows and dotted lines represented the residues of HAD hydrogels. Scale bars = 50 μm.

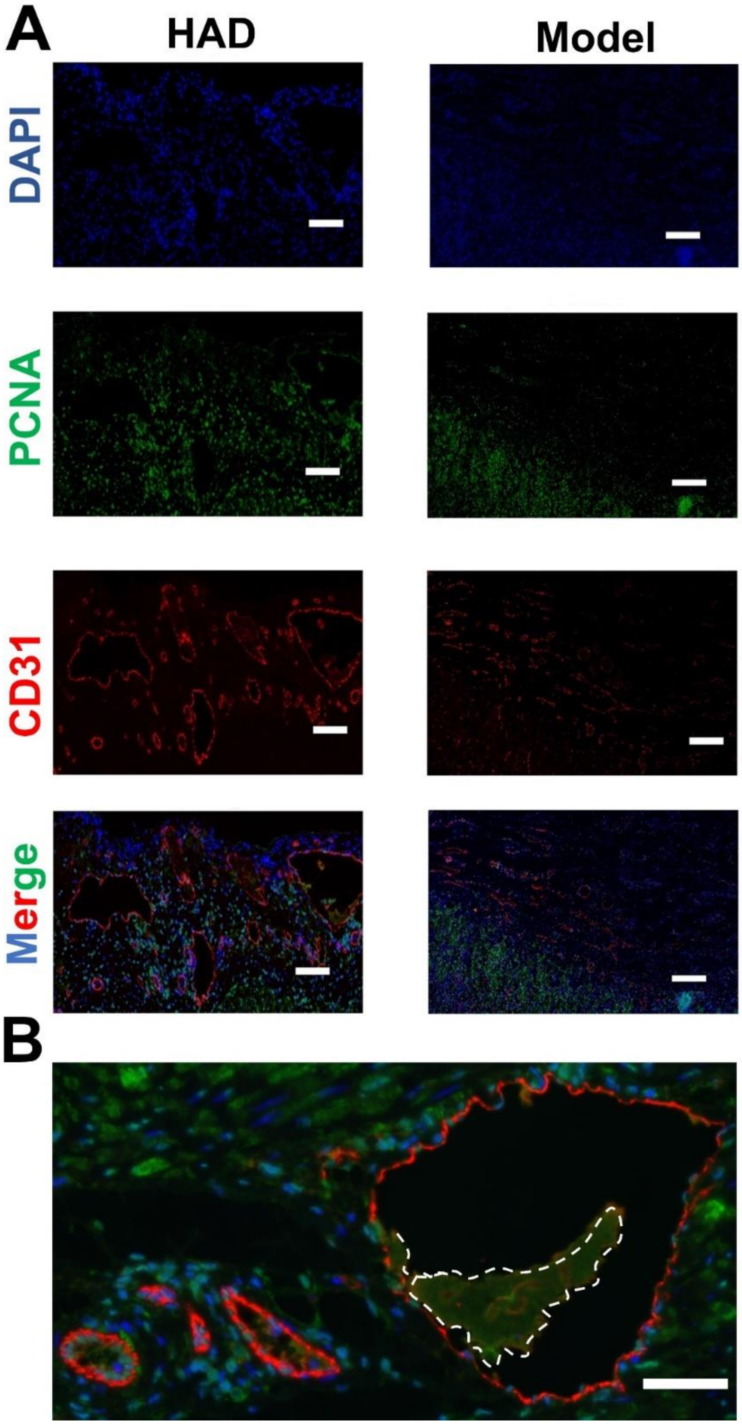

Figure S23. (A) Immunofluorescence staining of CD31 (red) and PCNA (green) in the model group and HAD group on day 7 after surgery. Scale bars = 50 μm. **(B)** Significant neovascularization was observed around the HAD degradation fragments after 7 days (400x magnification of the gastric perforation site). The white dotted line represented the residues of HAD hydrogels. Scale bars = 20 μm.

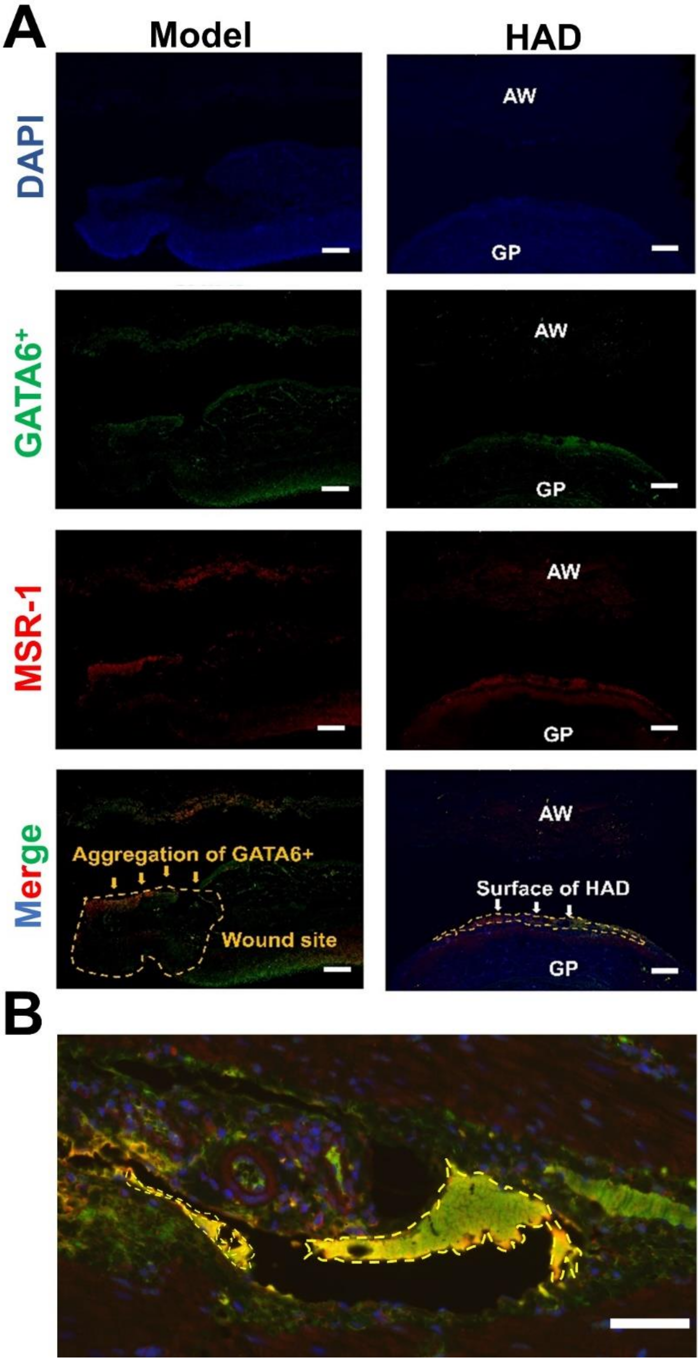

Figure S24. (A) Immunofluorescence staining of MSR-1 (red) and GATA6$^+$ macrophages (green) in the model group and HAD group on day 7 after surgery. Scale bars = 1000 μm. **(B)** No aggregation of floating GATA6+ macrophages was observed around the HAD degradation fragments after 7 days (400x magnification of the gastric perforation site). The yellow dotted line represented the residues of HAD hydrogels. Scale bars = 20 μm.

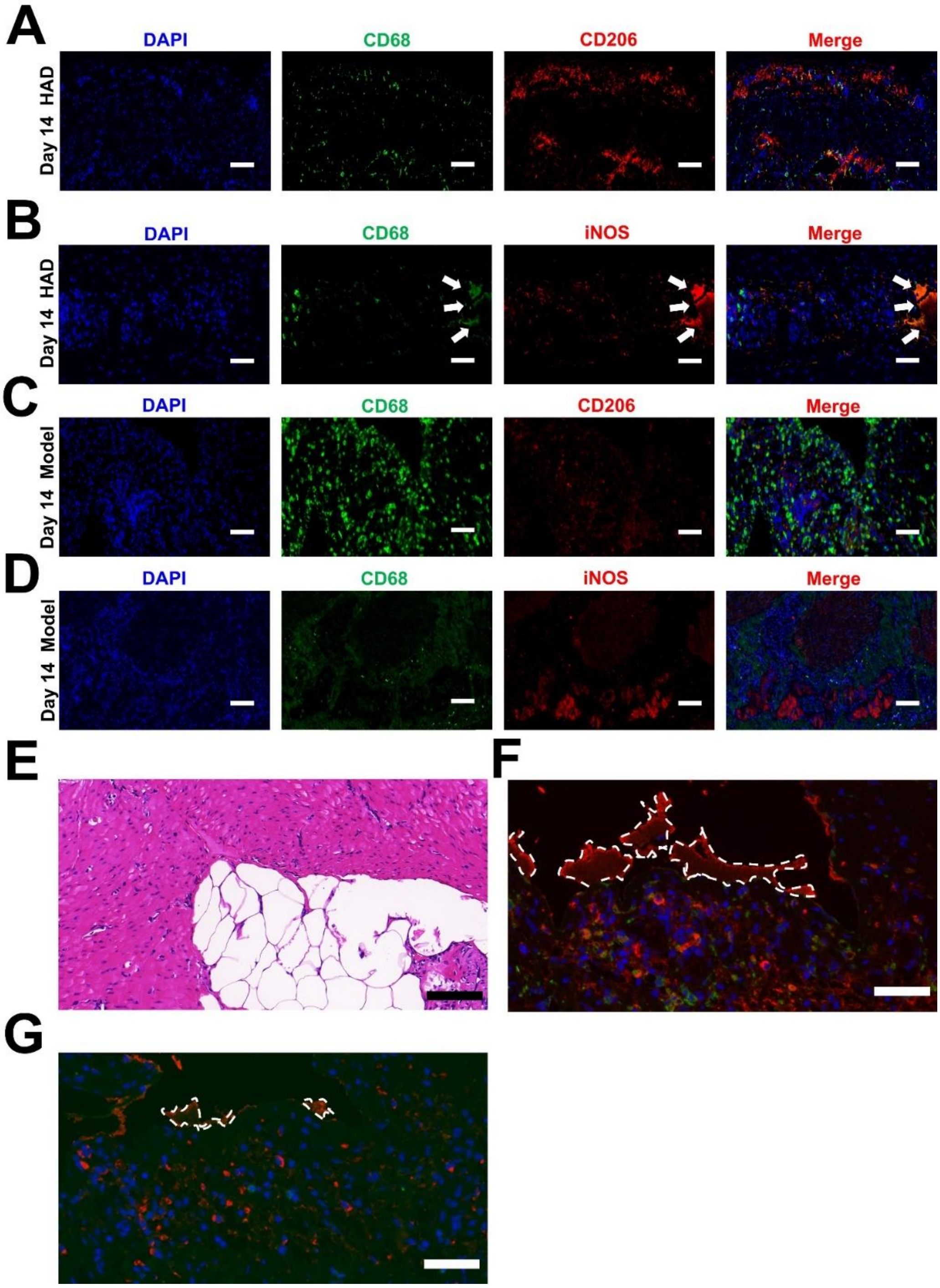

Figure S25. (A) Immunofluorescence staining of CD206 (red) and CD68 (green) in HAD group on day 14 after surgery. (**B)** Immunofluorescence staining of iNOS (red) and CD68 (green) in HAD group on day 14 after surgery. (**C)** Immunofluorescence staining of CD206 (red) and CD68 (green) in the Model group on day 14 after surgery. (**D)** Immunofluorescence staining of iNOS (red) and CD68 (green) in the Model group on day 14 after surgery. **(E)** In vivo HAD degradation fragments after 14 days did not

cause inflammatory cell infiltration in the muscular layer of the gastric perforation (400x magnification of the gastric perforation site). **(F)** Following 14 days of in vivo degradation, HAD induced M2 macrophage polarization (CD68 labeled in green, CD206 labeled in red, 400x magnification of the gastric perforation site). **(G)** Degree of M1 macrophage polarization promoted by HAD after 14 days of in vivo degradation. (CD68 labeled in green, iNOS labeled in red; 400x magnification of the gastric perforation site). The white arrows and dotted lines represented the residues of HAD hydrogels. Scale bars = 50 μm.

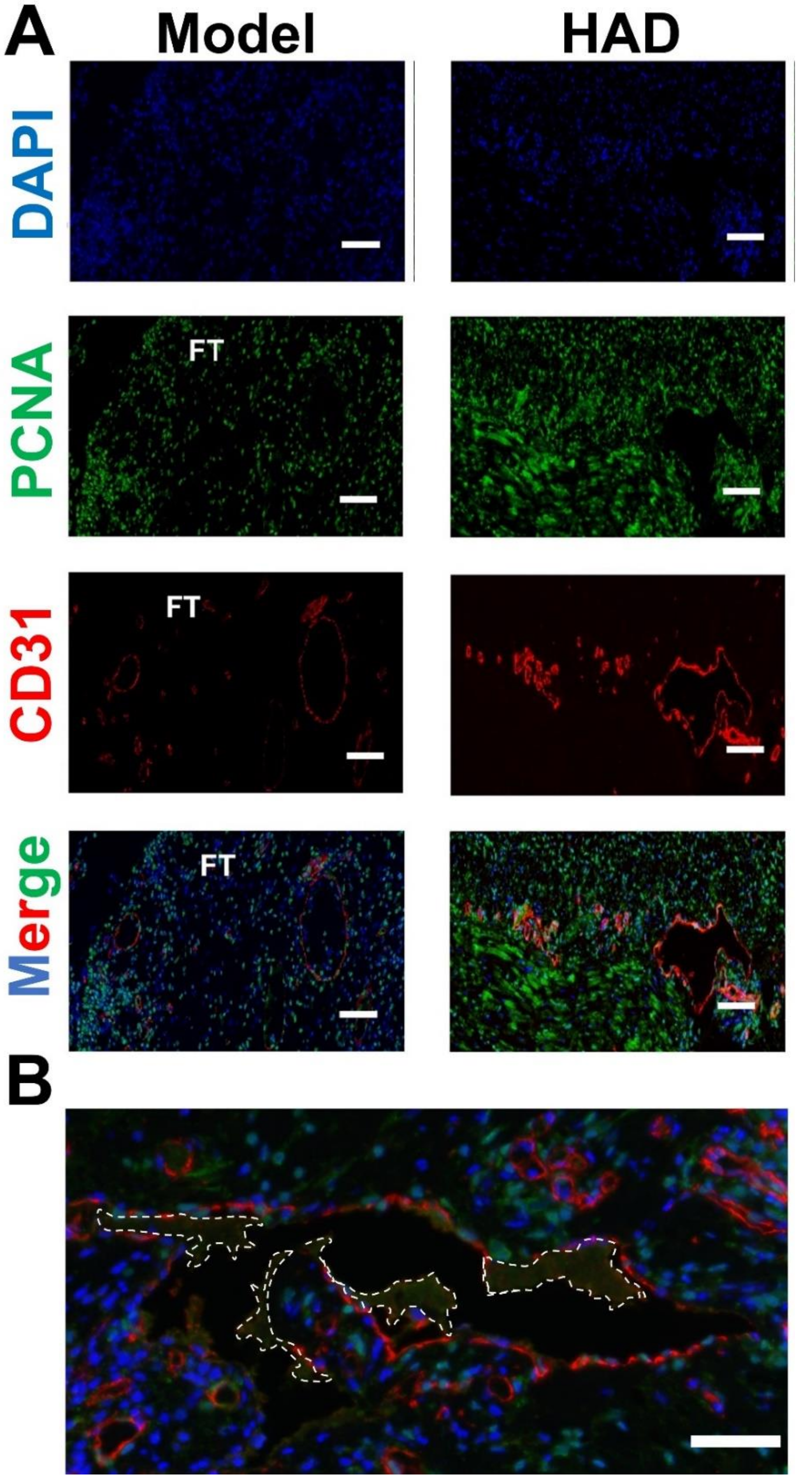

Figure S26. (A) Immunofluorescence staining of CD31 (red) and PCNA (green) in the model group and HAD group on day 14 after surgery. Scale bars = 50 μm. **(B)** Significant neovascularization was observed around the HAD degradation fragments after 14 days (400x magnification of the gastric perforation site). The white dotted line represented the residues of HAD hydrogels. Scale bars = 20 μm.

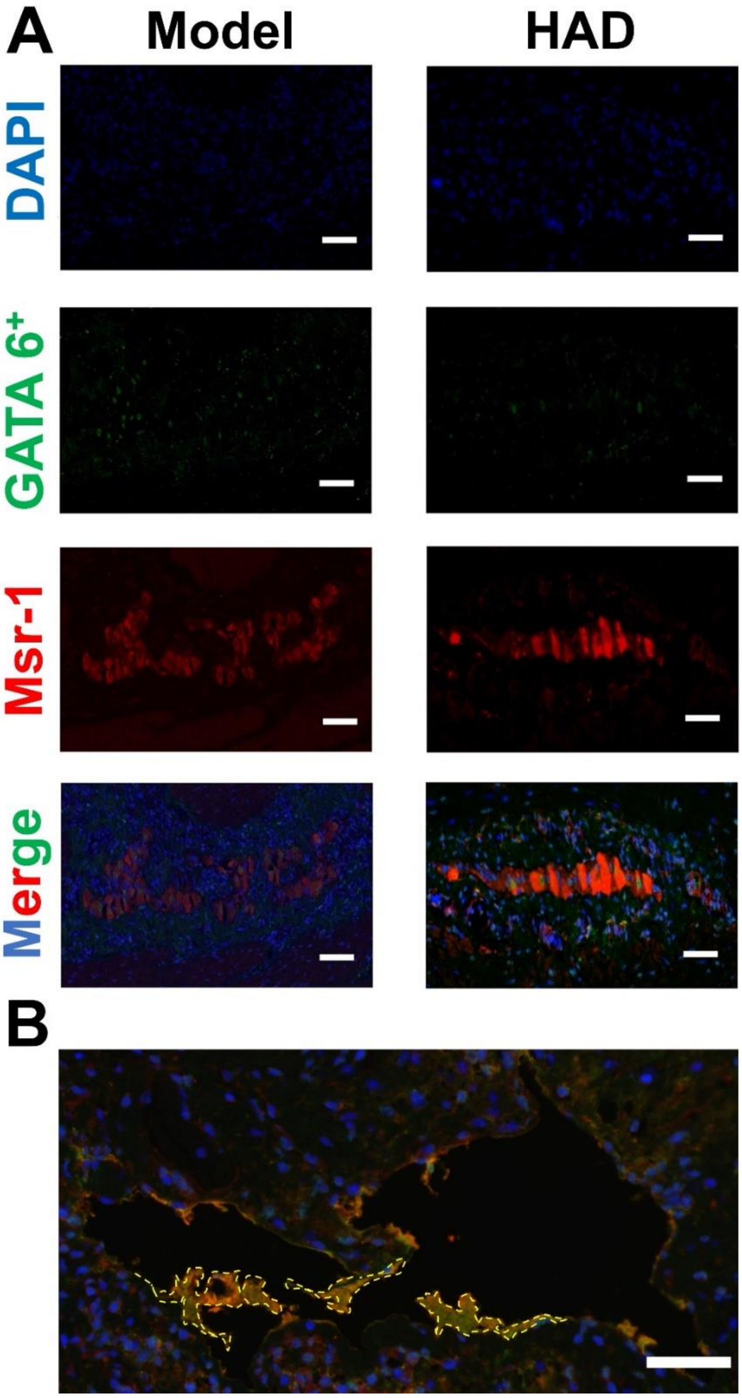

Figure S27. (A) Immunofluorescence staining of MSR-1 (red) and GATA6+ macrophages (green) in the model group and HAD group on day 14 after surgery. Scale bars = 50 μm. **(B)** No aggregation of floating GATA6+ macrophages was observed around the HAD degradation fragments after 14 days (400x magnification of the gastric perforation site). The yellow dotted line represented the residues of HAD hydrogels. Scale bars = 20 μm.

Supplementary Video 1. The burst pressure test of rat stomach.

Supplementary Video 2. Bursting pressure test of ex vivo porcine stomach with a 5-mm-diameter defect sealed by surgical sutures and HAD hydrogels.

Supplementary Video 3. Process of repairing the gastric operation of rabbits with HAD hydrogels through the laparoscopic surgery.

Supplementary Video 4. Simulated laparoscopic surgery of using HAD hydrogels to repair operations with the minimally invasive integrated device (MID).

Supplementary Video 5. The practical use of the minimally invasive integrated device (MID) in sealing gastric defects in the human specimen.

Supplementary Video 6. Process of repairing the micro intraoperative gastric operations using the MID and the postoperative laparoscopic exploration.

Supplementary Video 7. Process of achieving the rapid and robust hemostasis of the acute gastric operation bleeding using the MID in laparoscopy.

Supplementary Video 8. 2-week postoperative laparoscopic surveillance.

References

[1] J. Yu, Y. Qin, Y. Yang, X. Zhao, Z. Zhang, Q. Zhang, Y. Su, Y. Zhang, Y. Cheng, Robust hydrogel adhesives for emergency rescue and gastric perforation repair, Bioactive Materials 19 (2023) 703-716.

[2] K. Liu, H. Yang, G. Huang, A. Shi, Q. Lu, S. Wang, W. Qiao, H. Wang, M. Ke, H. Ding, T. Li, Y. Zhang, J. Yu, B. Ren, R. Wang, K. Wang, H. Feng, Z. Suo, J. Tang, Y. Lv, Adhesive anastomosis for organ transplantation, Bioactive Materials 13 (2022) 260-268.

[3] J. Chen, J.S. Caserto, I. Ang, K. Shariati, J. Webb, B. Wang, X. Wang, N. Bouklas, M. Ma, An adhesive and resilient hydrogel for the sealing and treatment of gastric perforation, Bioactive Materials 14 (2022) 52-60.

[4] C. Cui, T. Wu, X. Chen, Y. Liu, Y. Li, Z. Xu, C. Fan, W. Liu, A Janus Hydrogel Wet Adhesive for Internal Tissue Repair and Anti-Postoperative Adhesion, Advanced Functional Materials 30(49) (2020) 2005689.

[5] X. Wu, W. Guo, L. Wang, Y. Xu, Z. Wang, Y. Yang, L. Yu, J. Huang, Y. Li, H. Zhang, Y. Wu, G. Li, W. Huang, An Injectable Asymmetric-Adhesive Hydrogel as a GATA6+ Cavity Macrophage Trap to Prevent the Formation of Postoperative Adhesions after Minimally Invasive Surgery, Advanced Functional Materials 32(9) (2022) 2110066.

[6] X. Chen, J. Zhang, G. Chen, Y. Xue, J. Zhang, X. Liang, I.M. Lei, J. Lin, B.B. Xu, J. Liu, Hydrogel Bioadhesives with Extreme Acid-Tolerance for Gastric Perforation Repairing, Advanced Functional Materials 32(29) (2022) 2202285.

[7] Y. Jia, J. Feng, Z. Feng, J. Liu, Y. Yang, X. Li, M. Lei, H. Guo, Z. Wei, Y. Lv, F. Xu, An endoscopically compatible fast-gelation powder forms Janus-adhesive hydrogel barrier to prevent

postoperative adhesions, Proceedings of the National Academy of Sciences 120(6) (2023) e2219024120.

[8] W. Liang, W. He, R. Huang, Y. Tang, S. Li, B. Zheng, Y. Lin, Y. Lu, H. Wang, D. Wu, Peritoneum-Inspired Janus Porous Hydrogel with Anti-Deformation, Anti-Adhesion, and Pro-Healing Characteristics for Abdominal Wall Defect Treatment, Adv Mater 34(15) (2022) e2108992.

[9] B. Liu, Y. Kong, O.A. Alimi, M.A. Kuss, H. Tu, W. Hu, A. Rafay, K. Vikas, W. Shi, M. Lerner, W.L. Berry, Y. Li, M.A. Carlson, B. Duan, Multifunctional Microgel-Based Cream Hydrogels for Postoperative Abdominal Adhesion Prevention, ACS Nano 17(4) (2023) 3847-3864.
